# Methanol-induced transcription factor Mpp1 regulates the coordinated expression of multiple genes to achieve a balanced C1 metabolism in the methylotrophic yeast *Candida boidinii*

**DOI:** 10.1128/spectrum.02853-24

**Published:** 2025-03-31

**Authors:** Koichi Inoue, Nono Saso, Kosuke Iwase, Zhenyu Zhai, Takahiro Tsuji, Kazuyoshi Tabata, Rie Sano, Hiroya Yurimoto, Yasuyoshi Sakai

**Affiliations:** 1 Division of Applied Life Sciences, Graduate School of Agriculture, Kyoto University, Kitashirakawa-Oiwake, Sakyo-ku, Kyoto, Japan; The Hebrew University-Hadassah, Jerusalem, Israel

**Keywords:** methylotrophic yeast, transcription factor, concentration-regulated methanol induction, C1 metabolism, formaldehyde

## Abstract

Methylotrophic yeasts can grow on methanol as the sole carbon and energy source. They have been used as hosts for heterologous protein production, which is achieved by their strongly and tightly regulated methanol-induced gene promoters. The expression levels of methanol-induced genes, such as *AOD1* and *DAS1*, are regulated by concentration-regulated methanol induction (CRMI). In this study, we identified CbMpp1 as a methanol-induced transcription factor necessary for the expression of multiple genes in the methylotrophic yeast *Candida boidinii* and identified the upstream activating sequences in the *CbMPP1* promoter. The expression of *CbMPP1* was induced by methanol under the control of CRMI and was dependent on other transcription factors, CbTrm1, CbTrm2, and CbHap complex but not on CbMpp1 itself. To investigate the physiological significance of the CRMI-dependent expression of *CbMPP1*, we constructed strains overexpressing or constitutively expressing *CbMPP1* with varying copy numbers. Analysis of growth with methanol and transcript levels of methanol-induced genes of these strains revealed that an appropriate amount of CbMpp1 was required for optimal growth with methanol and that excessive amounts of CbMpp1 repressed the expression of methanol-inducible genes. Such abnormal expression of *CbMPP1* in these strains caused accumulation of formaldehyde and growth defects on methanol. In addition, the *Komagataella phaffii* strain constitutively expressing *KpMIT1* (a homolog of *CbMPP1*) at low levels exhibited retarded growth with methanol. Our results demonstrate that CbMpp1 and KpMit1, whose expression is regulated by CRMI, regulate the coordinated expression of multiple methanol-induced genes to achieve a balanced one-carbon metabolism.

**IMPORTANCE:**

Elucidating the molecular mechanism of how methanol-induced gene expression is regulated to attain a balanced one-carbon (C1) metabolism is important for determining the efficiency of heterologous protein production by methylotrophic yeasts. Although transcription factors involved in methanol-induced gene expression in these yeasts have been studied extensively, how these transcription factors regulate the expression of multiple methanol-induced genes in a concentration-regulated methanol induction (CRMI)-dependent manner has been unclear. In this study, we focused on the physiological significance of methanol inducibility of *CbMpp1* and *KpMit1* and found that CRMI-dependent *CbMPP1* and *KpMIT1* expressions are important for the coordinated regulation of multiple methanol-induced genes to facilitate a balanced C1 metabolism in the methylotrophic yeast.

## INTRODUCTION

Methylotrophic yeasts, such as *Komagataella phaffii* (synonym *Pichia pastoris*), *Ogataea polymorpha* (synonym *Hansenula polymorpha*), and *Candida boidinii*, are capable of growing on methanol as the sole carbon and energy source. They have been used as a host for heterologous gene expression using strong and tightly regulated methanol-induced gene promoters (1–5). In particular, *C. boidinii* was reported as the first example of methanol-utilizing yeast in 1969 (6), and its heterologous protein production system has been well established (7).

During growth with methanol, methylotrophic yeasts develop large peroxisomes containing substantial amounts of methanol-metabolizing enzymes such as alcohol oxidase (AOD) and dihydroxyacetone synthase (DAS) (Fig. S1). Cytosolic enzymes such as glutathione-dependent formaldehyde dehydrogenase (FLD), *S*-formylglutathione hydrolase, and formate dehydrogenase (FDH) are also significantly induced. The unique one-carbon (C1) metabolism in these yeasts and the related enzymes have been previously described (8–10). Methanol is first oxidized by AOD to generate formaldehyde and H_2_O_2_. Formaldehyde, a central intermediate of the methanol metabolism pathway, is positioned at the branch point between the assimilatory and dissimilatory pathways. In the assimilatory pathway, formaldehyde is fixed by DAS to xylulose 5-phosphate to produce dihydroxyacetone and glyceraldehyde 3-phosphate, which are then consumed to synthesize cell components. In the dissimilatory pathway, formaldehyde is further oxidized to CO_2_ by the glutathione-dependent formaldehyde oxidation pathway.

Expression of genes encoding these methanol-metabolizing enzymes is tightly regulated depending on the carbon source and strongly induced by methanol (11, 12). The gene promoters of these enzymes are substantially induced in the presence of methanol depending on the methanol concentration. The transcript levels of AOD- and DAS-encoding genes increase in the presence of 0.001%–0.1% methanol but decrease in the presence of more than 0.1% methanol, which has been reported as concentration-regulated methanol induction (CRMI) in *K. phaffii* (13). CRMI is responsible for regulating the metabolic flux of formaldehyde generated from the oxidation of methanol by AOD and also plays a key role in adapting to the change in methanol concentration in nature, e.g., on the leaf surface where methanol concentration oscillates diurnally (13, 14).

Unbalanced C1 metabolism is considered to result in excessive accumulation of formaldehyde, which is toxic to the cells (12, 15). Therefore, the expression levels of the formaldehyde-generating enzyme AOD and formaldehyde-consuming enzymes DAS and FLD should be properly controlled with respect to environmental methanol concentrations. Our previous methanol-limited chemostat culture experiment revealed that AOD activity decreased, but FLD activity increased with the increase in the dilution rate (16), which means the levels of AOD and FLD are regulated depending on methanol concentration to maintain the balance of C1 metabolism. With respect to the regulation of AOD and DAS, we have reported that induction of DAS preceded that of AOD during the early stages of methanol induction in *C. boidinii* (17), and that the promoter activity of *DAS1* is stronger than that of *AOD1* (18). More rapid and stronger expression of *DAS1* than *AOD1* might help to minimize the toxicity of formaldehyde in the peroxisome. Thus, methanol-metabolizing enzymes are not induced all at once, but multiple methanol-induced genes are coordinately regulated for the balanced C1 metabolism to avoid an accumulation of formaldehyde, the toxic intermediate.

Several transcription factors have been identified for methanol-induced gene expression in *K. phaffii*, *C. boidinii,* and *O. polymorpha*. The transcription factor *C. boidinii* Trm2 (CbTrm2), which is a homolog of *Saccharomyces cerevisiae* Adr1 and *K. phaffii* Mxr1 (KpMxr1), is involved in derepression (19, 20). The transcription factors, OpMpp1 and its homolog KpMit1 (21, 22), CbTrm1 and its homolog KpTrm1 (23, 24), and CbHap complex (25, 26), are involved in methanol induction and regulate the expression of many genes involved in methanol metabolism. Among the transcription factors related to methanol-induced gene expression in *K. phaffii* (13), KpMxr1 and KpMit1 were found to be necessary for CRMI of *AOX1* and *DAS1*. Zn(II)_2_Cys_6_-type OpMpp1 and KpMit1 are reported to be induced by methanol (21, 22). OpMpp1 regulates the expression of various proteins involved in methanol metabolism and peroxisome biogenesis (peroxins) in *O. polymorpha* (21). KpMit1 regulates many methanol-induced genes but does not participate in peroxisome proliferation and transportation of peroxisomal proteins (22). KpMit1, KpMxr1, and KpTrm1 were reported to bind to the promoter region of *AOX1* at different sites and do not interact with each other, and these transcription factors are speculated to regulate the expression of *AOX1* in a cooperative manner through a cascade in *K. phaffii* (22). However, the physiological significance of methanol inducibility of these Mpp1 homologs (OpMpp1 and KpMit1) has not been elucidated yet.

Methylotrophic yeasts sense the methanol concentration in their growth environments and regulate the expression level of methanol-induced genes. We reported that the cell surface membrane-spanning sensor Wsc family proteins KpWsc1 and KpWsc3 function in sensing the environmental concentration of methanol and are responsible for CRMI in *K. phaffii* (15). Furthermore, our previous studies have revealed that KpMxr1 receives the methanol signal from KpWsc1/3 and that the phosphoregulation of KpMxr1 plays a crucial role in CRMI (13). Because CRMI is considered to be one of the strategies to avoid unbalanced C1 metabolism, transcription factors involved in methanol-induced gene expression are supposed to regulate the proper expression of each methanol-induced gene. But the mechanism of this regulation of CRMI by these transcription factors has not been elucidated yet.

In this study, we identified the Zn(II)_2_Cys_6_-type zinc finger transcription factor CbMpp1 as a homolog of OpMpp1 and KpMit1 and focused on its physiological role in CRMI. We found that the expression of *CbMPP1* is regulated by CRMI and requires other transcription factors, CbTrm1, CbTrm2, and CbHap complex. We also found that an appropriate amount of CbMpp1 at an appropriate period of methanol induction is required for optimum growth with methanol using strains overexpressing or constitutively expressing *CbMPP1*. Additionally, similar experiments in *K. phaffii* revealed that an appropriate level of KpMit1 is sufficient for optimal growth with methanol. Our results show that CRMI-dependent CbMpp1 and KpMit1 regulate the coordinated expression and CRMI of multiple methanol-induced genes facilitating balanced C1 metabolism in the methylotrophic yeast.

## RESULTS

### Identification and gene expression of *CbMPP1*

A search of the *C. boidinii* draft genome sequence revealed the presence of the putative Zn(II)_2_Cys_6_ -type transcription factor CbMpp1, which is a homolog of OpMpp1 and KpMit1 (Fig. S2). CbMpp1 consists of 1,025 amino acids including the conserved motif for binding with DNA (76–132 a.a. from N-terminus). *CbMPP1* gene was localized at 3.4 kbp downstream of *DAS1* gene in *C. boidinii* genome, which is similar to the gene organization in *O. polymorpha* (21).

**Fig 1:**
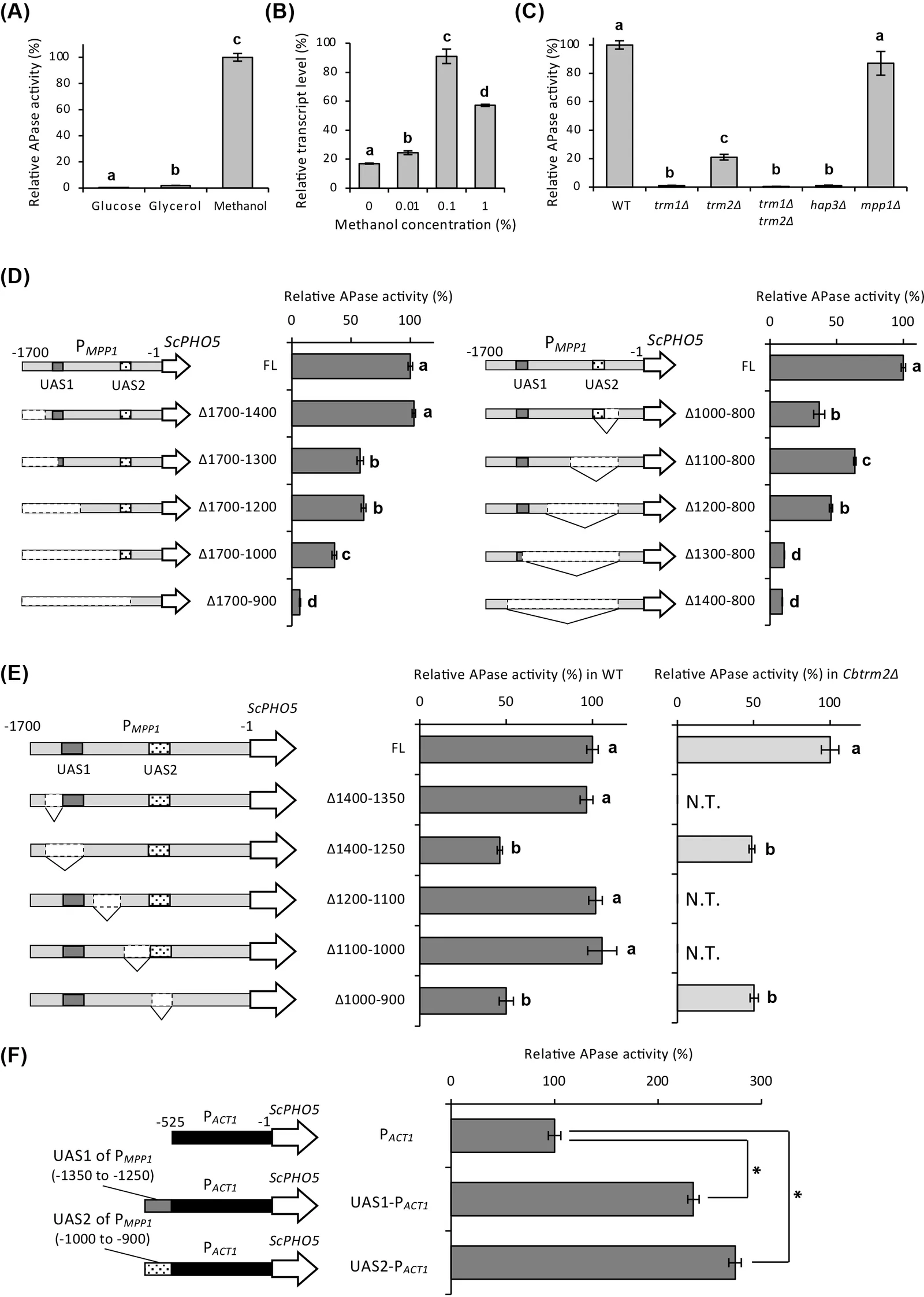
The expression of the *CbMPP1* promoter and its upstream activating sequences (UASs). (A) APase reporter activity under the control of P*_MPP1_*. Cells were cultured in YNB media containing 2% glucose, 2% glycerol, or 0.7% methanol for 8 h. APase activities are shown relative to that of methanol-grown cells (224 ± 6.51 U/OD_610_). (B) Transcript level of *CbMPP1* depending on methanol concentration in the wild-type strain. Total mRNA was prepared from the cells of each strain cultured in YNB media containing 0%, 0.01%, 0.1%, and 1% methanol for 2 h. The transcript levels were normalized using *CbACT1* as the reference standard. Relative transcript levels are shown compared to that of the glucose-cultured sample. (C) APase reporter activity under the control of P*_MPP1_* in various gene-disrupted strains. Cells were cultured in YNB medium containing 0.7% methanol for 8 h. APase activities are shown relative to that of the wild-type (WT) strain (224 ± 6.51 U/OD_610_). (D) APase reporter activity of truncated *CbMPP1* promoters (P*_MPP1_*) deleted from 5´ to 3´ (left) and from 3´ to 5´ (right). Highlighted region in the promoter means putative UASs estimated from the results of APase assay using truncated P*_MPP1_*. APase activities are shown relative to that in the control strain possessing full-length (FL) P*_MPP1_* (129 ± 2.49 U/OD_610_ for left panel and 113 ± 1.81 U/OD_610_ for right panel). (E) The activity of partially truncated P*_MPP1_* in the wild-type strain (gray bars) and *Cbtrm2*Δ strain (black bars). Relative activity levels compared to that in the control strain possessing full-length (FL) P*_MPP1_* (149 ± 4.89 U/OD_610_ for WT and 64.1 ± 3.63 U/OD_610_ for the *Cbtrm2*Δ strain) are indicated. Putative upstream activating sequences UAS1 (from –1,350 to –1,250) and UAS2 (from –1,000 to –900) in P*_MPP1_* are indicated. N.T., not tested. (F) APase reporter activity of P*_ACT1_* attached to UAS1 and UAS2 in P*_MPP1_*. Cells were pre-cultured in YNB medium containing 2% glucose and shifted to YNB medium containing 0.7% methanol for 8 h. APase activities are shown relative to that in the control strain possessing P*_ACT1_* (31.5 ± 1.91 U/OD_610_). Error bars represent standard error values from three independent experiments. Groups indicated by different symbols showed statistically significant differences (e.g., between a and b, *: *P* < 0.05) as determined by one-way analysis of variance.

To investigate the regulation of *CbMPP1* expression by carbon sources, we performed a promoter activity assay using acid phosphatase (APase) as a reporter. The *CbMPP1* promoter (P*_MPP1_*) activity is completely repressed under glucose-culture condition, slightly upregulated under glycerol-culture condition, and strongly induced under methanol-culture condition (Fig. 1A). The transcript level of *CbMPP1* increased in the presence of 0.01%–0.1% methanol and exhibited the maximum level at 0.1%, but decreased in the presence of 1% methanol (Fig. 1B). These results demonstrate that *CbMPP1* is induced by methanol, similar to that of *OpMPP1* and *KpMIT1*, and that the expression of *CbMPP1* is regulated by CRMI in *C. boidinii*.

Next, we investigated the dependency of *CbMPP1* expression on transcription factors involved in methanol-induced gene expression. APase reporter activity under P*_MPP1_* was determined in various gene disruptants for transcription factors. P*_MPP1_* activity was lost in *Cbtrm1*Δ and *Cbhap3*Δ strains (Fig. 1C). APase reporter activity decreased to about 20% in the *Cbtrm2*Δ strain compared with the wild-type strain and was entirely lost in the *Cbtrm1*Δ*trm2*Δ strain (Fig. 1C). In contrast, disruption of *CbMPP1* did not affect its promoter activity (Fig. 1C). These results indicate that the methanol-induced expression of *CbMPP1* completely depends on the CbTrm1 and CbHap complex and partly depends on CbTrm2, but does not depend on CbMpp1 itself.

For further characterization of P*_MPP1_*, we identified the upstream activating sequences (UASs) in P*_MPP1_*. For these experiments, we constructed strains possessing a single copy of APase expression cassette with truncated P*_MPP1_* and confirmed the single-copy integration of the expression cassette by Southern blot analysis (Fig. S3A and B). The APase reporter activity of 5´ to 3´ truncated P*_MPP1_* demonstrated that the promoter regions from –1,400 to –1,300, from –1,200 to –1,000, and from –1,000 to –900 are important for its function (Fig. 1D, left panel). In the same way, the APase reporter activity of 3´ to 5´ truncated P*_MPP1_* indicated that the promoter regions from –1,000 to –800,–1,200 to –1,100, and –1,300 to –1,200 are crucial for its function (Fig. 1D, right panel). Furthermore, partial deletion analysis of putative UAS regions was performed to confirm their importance for P*_MPP1_* activity. The APase reporter activity decreased between PHOΔ1400–1350 and PHOΔ1400–1250, PHO-FL and PHOΔ1000–900 in the wild-type strain (Fig. 1E). This result suggests that the DNA regions from –1,350 to –1,250 (designated as UAS1) and –1,000 to –900 (designated as UAS2) are essential for P*_MPP1_* activity. Since P*_MPP1_* activity remained in the *Cbtrm2*Δ strain to about 20% of the wild-type strain (Fig. 1C), the *Cbtrm2*Δ strain was used for P*_MPP1_* deletion analysis to evaluate the dependency of CbTrm2 on UASs. The activity of P*_MPP1_* lacking these regions also decreased in the *Cbtrm2*Δ strain compared to that of the full-length P*_MPP1_* (FL) as in the case in the wild-type strain, indicating that these promoter regions are independent of CbTrm2 (Fig. 1E). Finally, to investigate their sufficiency for their promoter activity, UAS1 and UAS2 were fused to the *CbACT1* promoter (P*_ACT1_*), and their promoter activity was measured. *CbACT1* encodes actin, and its gene expression level is not significantly influenced by culture conditions. The APase reporter activity of UAS1-P*_ACT1_* and UAS2-P*_ACT1_* significantly increased under methanol-culture condition (Fig. 1F), showing that UAS1 and UAS2 are sufficient for methanol-induced promoter activity of P*_MPP1_*.

### The function of CbMpp1 during growth with methanol

**Fig 2:**
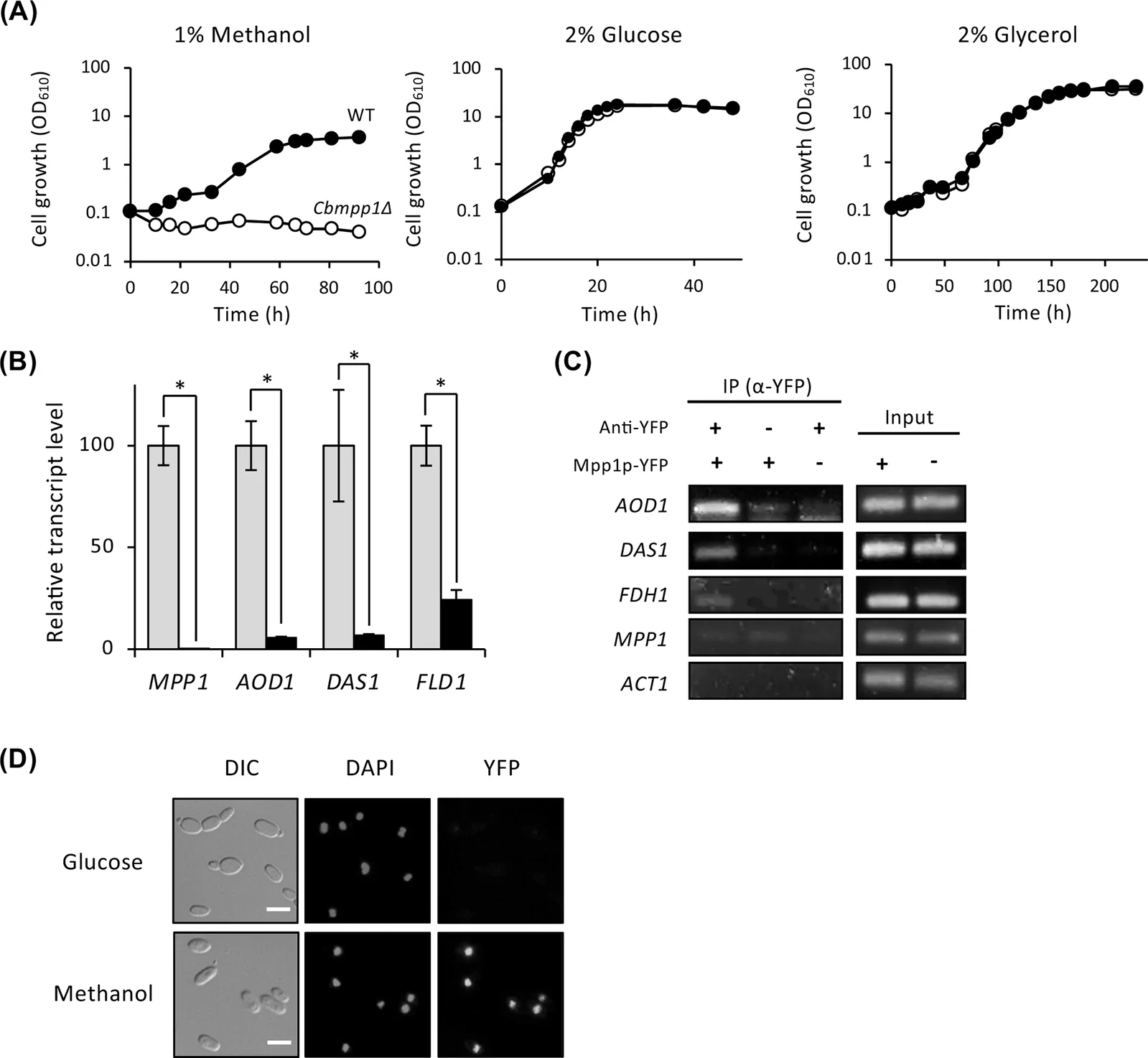
The function of CbMpp1 during the growth with methanol. (A) Cell growth of wild-type (filled circles) and *Cbmpp1*Δ (open circles) strains on various carbon sources. Cells were pre-cultured in YNB medium containing 2% glucose and then grown in YNB medium containing 2% glucose, 1% methanol, or 2% glycerol. (B) Relative transcript levels of *AOD1*, *DAS1*, *FLD1,* and *FDH1* in wild-type (gray bars) and *Cbmpp1*Δ (black bars) strains under methanol-culture condition. Cells were pre-cultured in YNB medium containing 2% glucose and shifted to YNB medium containing 0.7% methanol for 4 h. Error bars represent standard error values from three independent experiments. (C) Chromatin immunoprecipitation of CbMpp1-YFP with the DNA fragments of *AOD1*, *DAS1*, *FDH1, CbMPP1,* and *ACT1*. Cells were pre-cultured in YNB medium containing 2% glucose and shifted to YNB medium containing 0.7% methanol for 4 h. (D) Fluorescence microscopy of CbMpp1-YFP. Cells were shifted from YNB medium containing 2% glucose to YNB medium containing 0.5% methanol for 5 h. Subsequently, they were treated with 70% ethanol for 30 min and stained with 50 µg/L 4′,6-diamidino-2-phenylindole (DAPI) for 20 min. DAPI was used to stain the nucleus. DIC, differential interference contrast. The scale bars correspond to 5.0 µm.

Next, we examined the cell growth of wild-type and *Cbmpp1*Δ strains with various carbon sources. *Cbmpp1*Δ showed a significant growth defect under methanol-culture condition, while there is no difference compared to wild-type strain under glucose-, glycerol-, ethanol-, and oleate-culture conditions (Fig. 2A; Fig. S4A). *Cbmpp1*Δ also showed a decrease in *AOD1*, *DAS1,* and *FLD1* transcript levels under methanol-culture condition (Fig. 2B). These results reveal that CbMpp1 is necessary for cell growth with methanol and for methanol-induced gene expression. Chromatin immunoprecipitation analysis revealed that CbMpp1 interacted with the promoter regions of *AOD1*, *DAS1,* and *FDH1* (Fig. 2C). In contrast, CbMpp1 did not interact with the promoter regions of *CbMPP1* and *CbACT1* (Fig. 2C). These results suggest that CbMpp1 positively regulates the transcription of *AOD1*, *DAS1,* and *FDH1,* but does not exert an influence on the transcript level of *CbMPP1* itself.

Subcellular localization of CbMpp1-YFP was observed by fluorescent microscopy. No fluorescence of CbMpp1-YFP was observed under glucose-culture condition, while CbMpp1-YFP was localized to the nucleus under methanol-culture condition (Fig. 2D). These results demonstrated that CbMpp1 functions as a transcription factor in the nucleus and regulates the expression of multiple methanol-induced genes.

### Overexpression or constitutive expression of *CbMPP1* caused a growth defect on methanol

**Fig 3:**
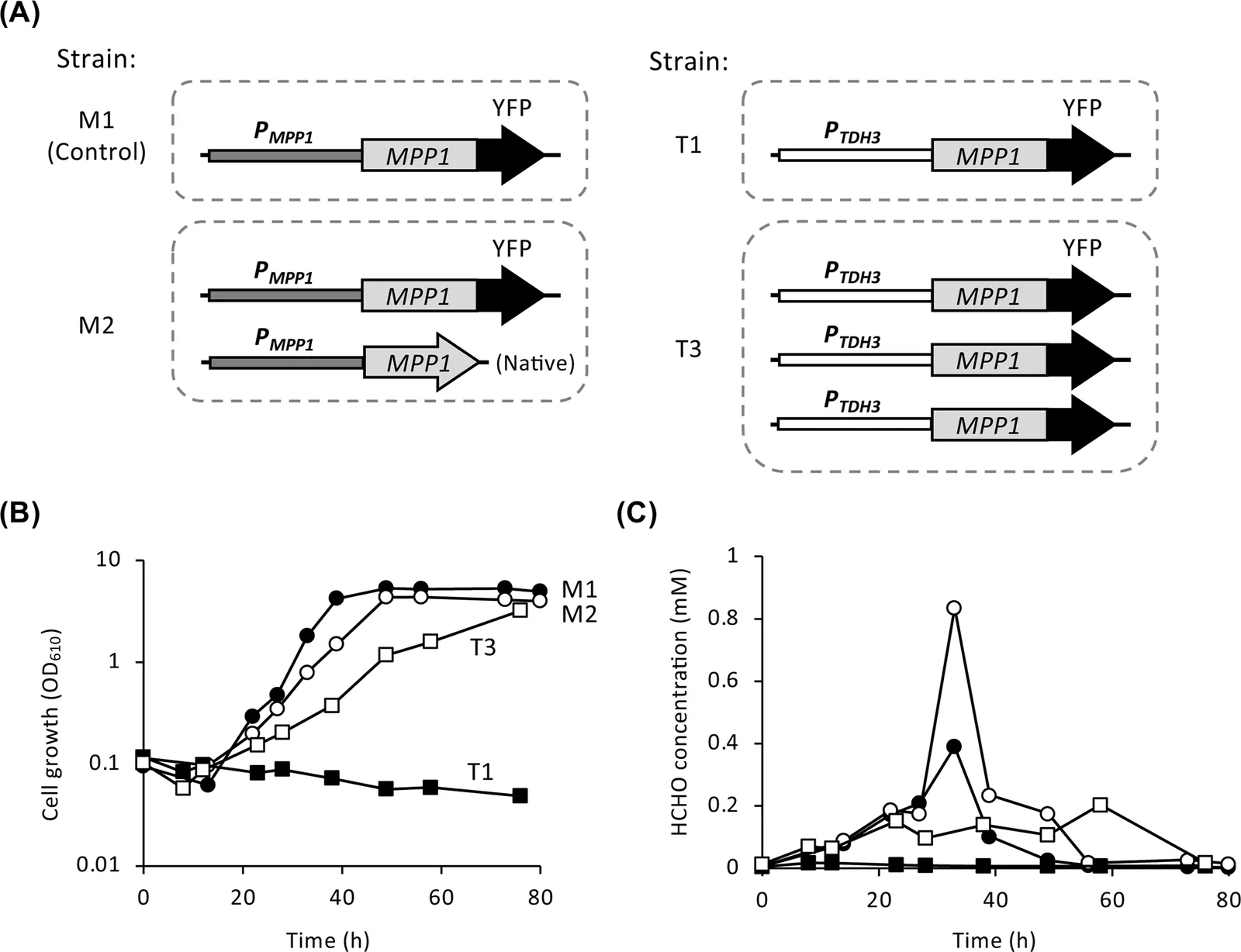
The effects of overexpression or constitutive expression of *CbMPP1* on cell growth and formaldehyde accumulation. (A) The model of the strains used in this experiment. The control strain M1 was derived from the *Cbmpp1*Δ strain and had one copy of P*_MPP1_-CbMPP1*-YFP. Strain M2 had one copy of P*_MPP1_-CbMPP1*-YFP in addition to the native *CbMPP1* gene. Strains T1 and T3 were derived from the *Cbmpp1*Δ strain and had one and three copies of P*_TDH3_-CbMPP1*-YFP. (B, C) Cell growth (B) and formaldehyde concentration (C) of strains M1 (filled circles), M2 (open circles), T1 (filled squares), and T3 (open squares) on YNB medium containing 1% methanol.

To investigate the physiological significance of methanol inducibility of *CbMPP1*, we constructed strains overexpressing or constitutively expressing *CbMPP1* (Fig. 3A). We used the *CbTDH3* promoter (P*_TDH3_*), which is constitutively activated regardless of carbon sources, for the constitutive expression of *CbMPP1*. The control strain M1 had a copy of the plasmid for expressing *CbMPP1*-YFP under the control of P*_MPP1_*. Strain M2 had a copy of P*_MPP1_-CbMPP1*-YFP (overexpression strain) in addition to the native *CbMPP1* gene. Strain T1 contained a copy of the plasmid for expressing *CbMPP1*-YFP under the control of P*_TDH3_*, and strain T3 had three copies of that plasmid (constitutive expression strains). The *Cbmpp1*Δ strain was used as a transformation host to construct strains M1, T1, and T3.

There was no difference in the growth of all strains on glucose or ethanol media (Fig. S4B). Under methanol-culture condition, strain M2 exhibited a slightly lower growth rate compared to strain M1 (Fig. 3B). Strain T3 showed a lower growth rate than strain M1 under methanol-culture condition, but strain T1 exhibited no growth at all. These results suggest that the single-copy expression of *CbMPP1* under P*_TDH3_* is not sufficient to have optimal growth ability on methanol, and overexpression (strain M2) or multi-copy constitutive (strain T3) expression of *CbMPP1* causes a decrease in growth rate. Formaldehyde concentration in the supernatant of culture medium was determined at the same time point of the growth measurement in these strains (Fig. 3C). In strain M1, the temporal increase of formaldehyde concentration (0.4 mM) was observed at 33 h and it immediately disappeared during further growth with methanol. On the other hand, the temporal increase of formaldehyde in strain M2 was more than twofold higher (~0.9 mM) than that in strain M1. Formaldehyde was not observed in strain T1, while 0.1 mM–0.2 mM formaldehyde accumulated in strain T3 for a long period of cultivation, and the consumption of formaldehyde was delayed. These results indicate that abnormal regulation of *CbMPP1* expression affects methanol metabolism and causes growth defects on methanol by surplus or prolonged accumulation of formaldehyde.

### Overexpression or constitutive expression of *CbMPP1* affects the transcript levels of multiple methanol-induced genes

**Fig 4:**
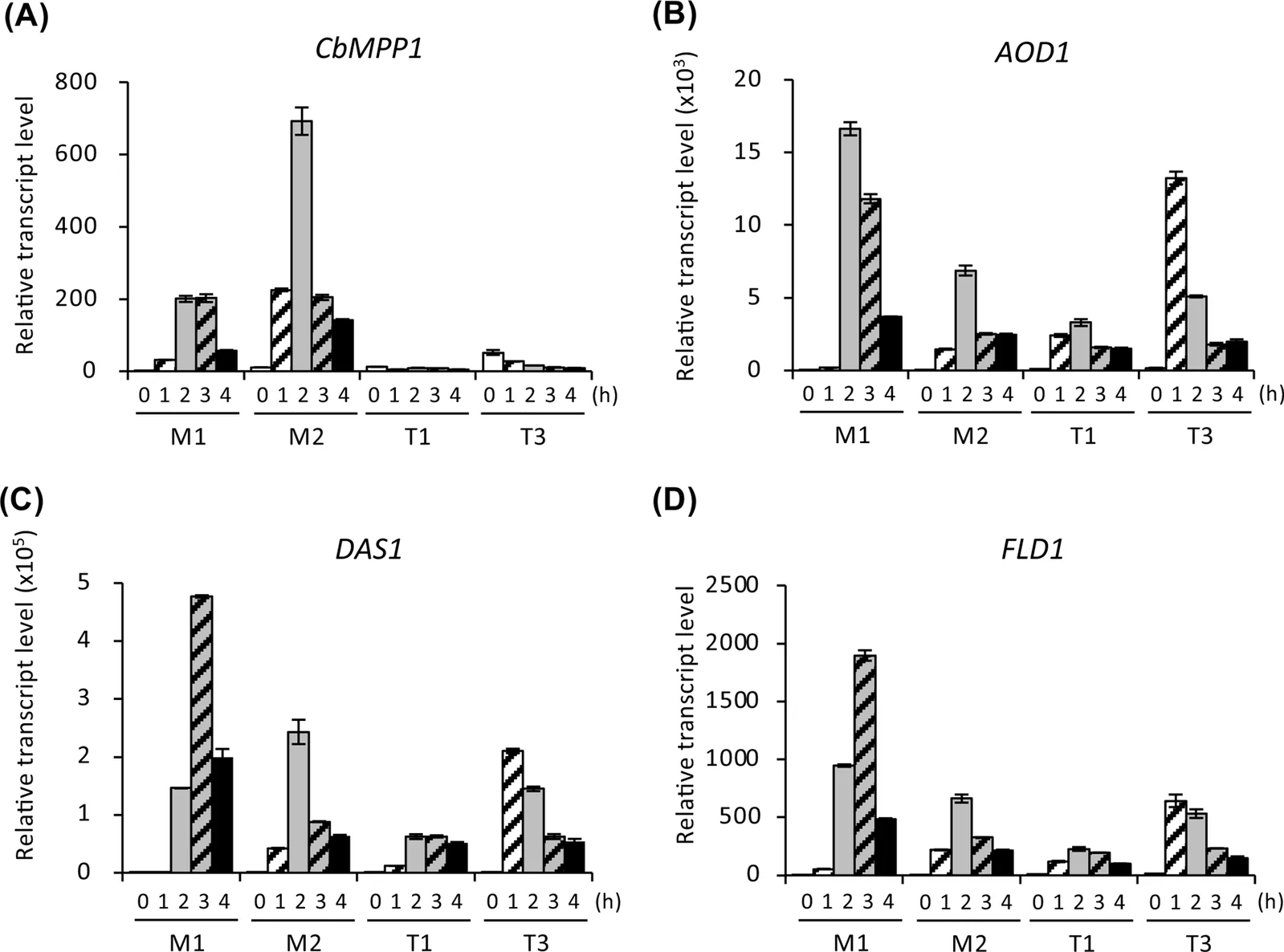
Transcript levels of methanol-induced genes in the strains overexpressing or constitutively expressing *CbMPP1* under methanol-culture condition. Total RNA was prepared from the cells of strains M1, M2, T1, or T3 cultured in YNB medium containing 0.7% methanol for 0 h (white bars), 1 h (white bars with diagonal line), 2 h (gray bars), 3 h (gray bars with diagonal line), and 4 h (black bars). Transcript levels of *CbMPP1* (A), *AOD1* (B), *DAS1* (C), and *FLD1* (D) in each strain were analyzed by quantitative reverse transcription PCR. The transcript levels were normalized using *CbACT1* as the reference standard. Relative transcript levels are shown compared to that of the 0 h sample in strain M1. Error bars represent standard error values from three independent experiments.

The effect of overexpression or constitutive expression of *CbMPP1* on the transcript levels of methanol-induced genes was analyzed using strains M1, M2, T1, and T3. The transcript level of *CbMPP1* in strain M2 under methanol-culture condition increased compared to that of strain M1 (Fig. 4A), which confirms overexpression of *CbMPP1*. In contrast, transcript levels of *AOD1*, *DAS1,* and *FLD1* in strain M2 were significantly lower than those in strain M1 (Fig. 4B through D). This result indicates that overexpression of *CbMPP1* reduces the expression level of multiple methanol-induced genes.

As shown in Fig. 4A, the transcript levels of *CbMPP1* in strains T1 and T3 were lower than the control strain M1 under methanol-culture condition. The *CbMPP1* transcript level in strain T3 was higher than those in strain T1 at all of the time points. These results confirmed the gene copy number-dependent constitutive expression of *CbMPP1* in strains T1 and T3. The transcript levels of *AOD1*, *DAS1,* and *FLD1* in strains T1 and T3 decreased under methanol culture-condition compared to strain M1, except in the case of strain T3 at 1 h (Fig. 4B through D). Strain T3 at 0 h, i.e., glucose-cultured cells, exhibited significant transcript levels of *CbMPP1* (Fig. 4A). This may cause the higher transcript levels of *AOD1*, *DAS1,* and *FLD1* in strain T3 at 1 h.

In addition, we analyzed the subcellular localization of CbMpp1 and protein levels of CbMpp1, AOD, and DAS in strains M1, T1, and T3. In strain M1, CbMpp1-YFP was not observed under glucose-culture condition, while it was localized to the nucleus under methanol-culture condition (Fig. S5A). In contrast, CbMpp1-YFP in strains T1 and T3 localized to the nucleus both under glucose- and methanol-culture conditions. These results suggest that CbMpp1-YFP can localize to the nucleus regardless of the carbon sources. Judging from immunoblot analysis, the CbMpp1-YFP protein in strains T1 and T3 was observed under glucose-culture condition and decreased during growth with methanol (Fig. S5B), which is consistent with the result of the transcript level analysis (Fig. 4A). CbMpp1 protein produced during growth with glucose seemed to be rapidly degraded by the medium shift to methanol (Fig. S5B). Therefore, *CbMPP1* gene expression under P*_MPP1_* appears to be necessary to keep the proper amount of CbMpp1. The protein levels of AOD and DAS in strains T1 and T3 were lower than those in strain M1 (Fig. S5B). In particular, DAS protein level in strain T1 was significantly low, suggesting that this could cause the growth defect of strain T1 on methanol.

### Proper regulation of *CbMPP1* is necessary for CRMI of multiple methanol-induced genes

**Fig 5:**
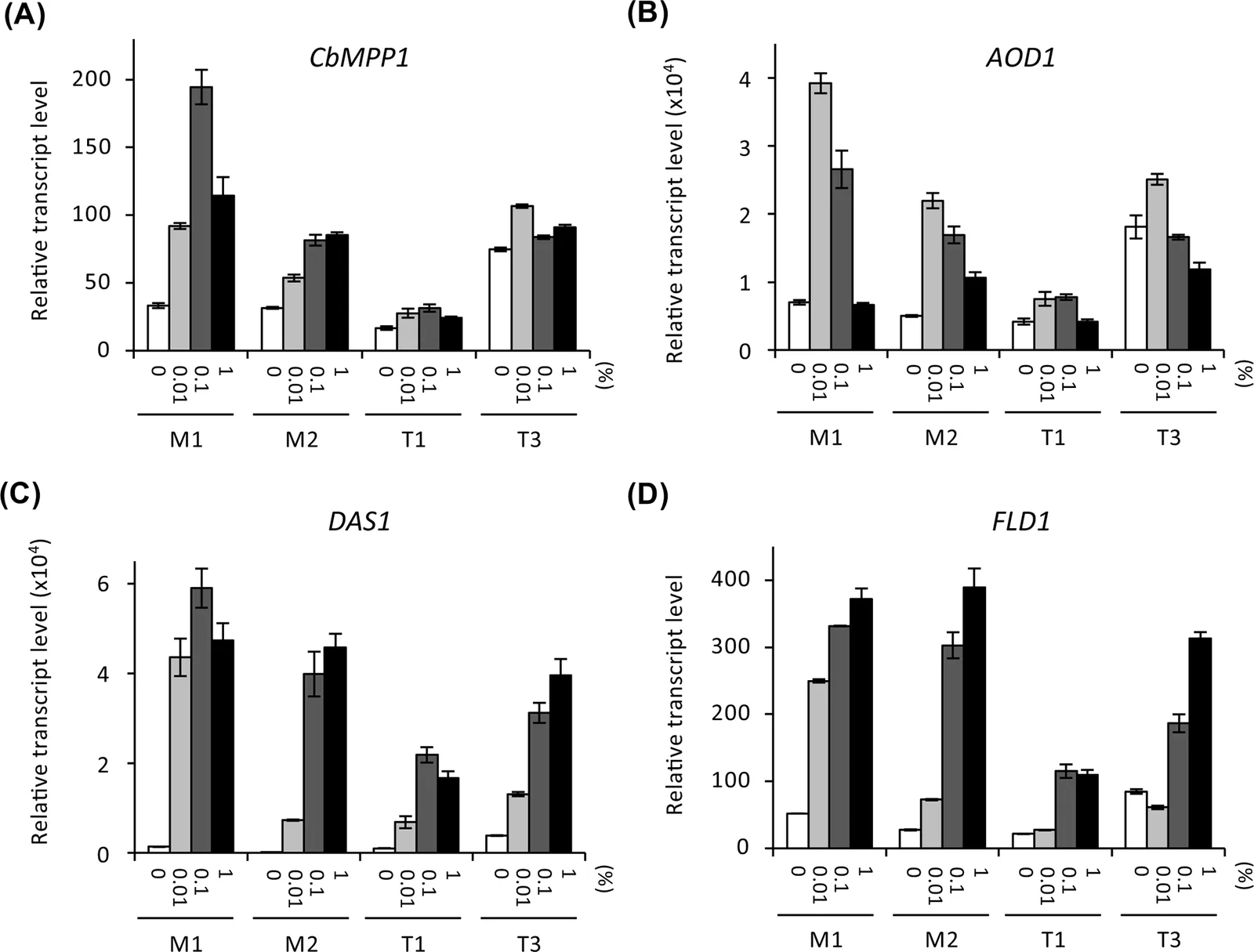
Transcript levels of *CbMPP1*, *AOD1*, *DAS1,* and *FLD1* in response to change in methanol concentration in strains M1, T1, T3, and M2. Total RNA was prepared from the cells of the strains cultured in YNB media containing 0% (white bars), 0.01% (light gray bars), 0.1% (dark gray bars), and 1% (black bars) methanol for 2 h. Transcript levels of *CbMPP1* (A), *AOD1* (B), *DAS1* (C), and *FLD1* (D) in each strain were analyzed by qRT-PCR. The transcript levels were normalized using *CbACT1* as the reference standard. Relative transcript levels are shown compared to that of the glucose-cultured sample in strain M1. Error bars represent standard error values from three independent experiments.

Next, we investigated the relationship between the regulation of *CbMPP1* expression and CRMI. Transcript levels of *CbMPP1*, *AOD1*, *DAS1,* and *FLD1* in strains M1, M2, T1, and T3 under various methanol concentrations were measured by quantitative reverse transcription PCR (qRT-PCR), and their dependency on methanol concentration was evaluated. In strain M1, the transcript levels of *CbMPP1*, *AOD1,* and *DAS1* increased in the presence of 0.01%–0.1% methanol but decreased in the presence of more than 0.1% methanol (Fig. 5A through C). Although the transcript level of *FLD1* increased in the presence of 0.01%–0.1% methanol, it was not repressed under 1% methanol-culture condition (Fig. 5D). Consistently, the transcript levels of *CbMPP1* in all methanol concentrations were lower in strains M2, T1, and T3 compared to strain M1 (Fig. 5A). Furthermore, a CRMI-dependent transcriptional activation and repression was not observed in *CbMPP1* expression of strains M2, T1, and T3 (Fig. 5A). The transcript level of *AOD1* and *DAS1* also decreased in strains M2, T1, and T3 compared to strain M1 (Fig. 5B and C). CRMI of *DAS1* was lost in strains M2 and T3 (Fig. 5C). These results suggest that CRMI of *CbMPP1* under the control of P*_MPP1_* plays a critical role in maintaining sufficient and proper regulation of multiple methanol-induced genes. In addition, constitutive or excessive expression of *CbMPP1* leads to impedance of CRMI. These results indicate that the expression of *CbMPP1* is tightly and accurately regulated for maintaining the proper methanol metabolism in *C. boidinii*.

### Regulation of *KpMIT1* plays a crucial role in CRMI of methanol-induced genes in *K. phaffii*

In our previous study, we reported that expression of *KpMIT1* is regulated in a CRMI-dependent manner in *K. phaffii* (13). We then investigated the effect of overexpression or constitutive expression of *KpMIT1* on the transcript levels of methanol-induced genes and on methanol metabolism in *K. phaffii*. Similarly to the experiments with *C. boidinii*, we constructed *K. phaffii* strains KpM1, KpM2, KpT1, and KpT2. Strains KpM1 and KpM2 harbor one and two copies of *KpMIT1* under the *KpMIT1* promoter, respectively, while strains KpT1 and KpT2 harbor one and two copies of *KpMIT1* under the *KpTDH3* promoter, respectively.

**Fig 6:**
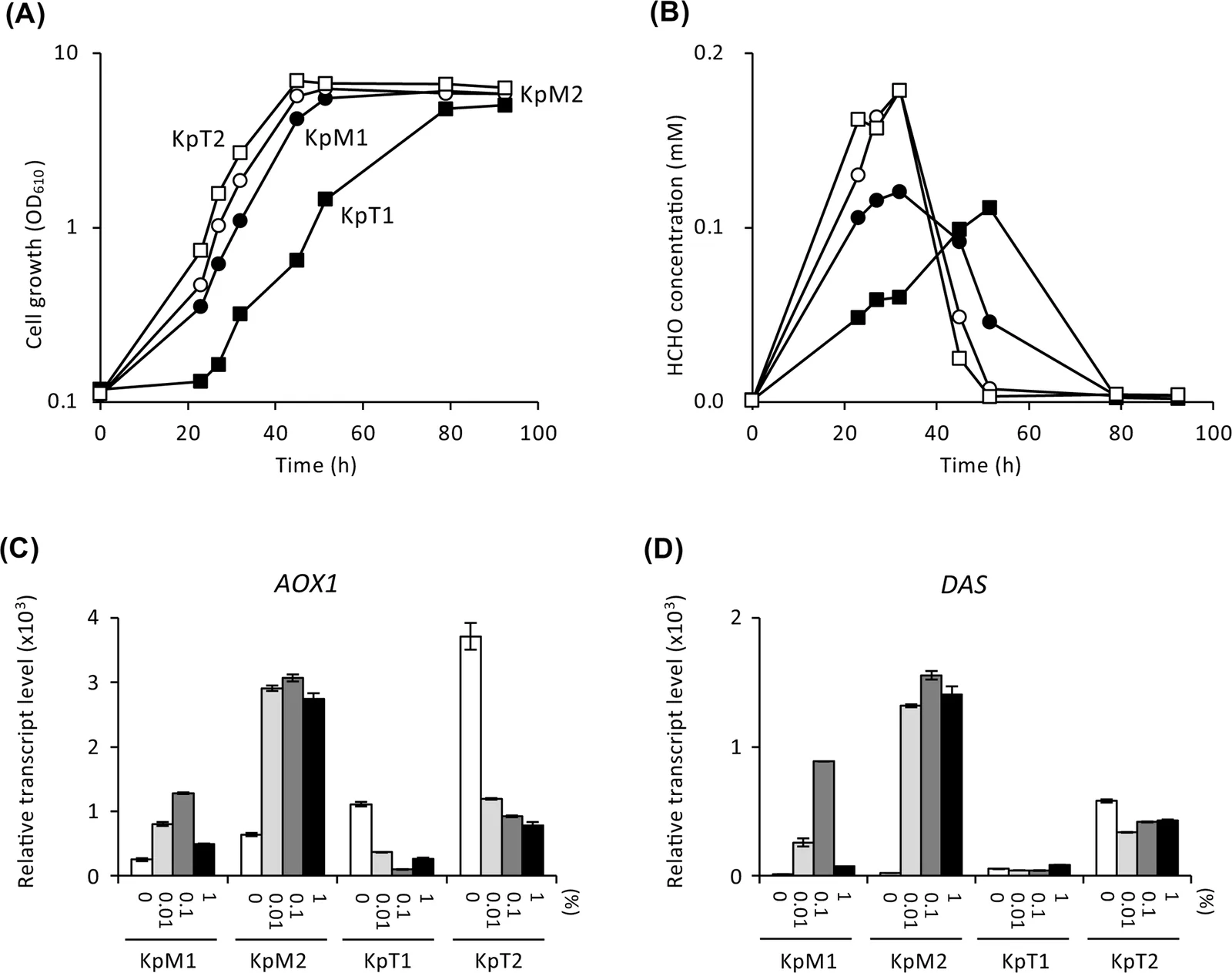
Cell growth (A) and formaldehyde concentration (B) of strains KpM1 (filled circles), KpM2 (open circles), KpT1 (filled squares), and KpT2 (open squares) on YNB medium containing 1% methanol. Transcript levels of *KpAOX1* (C) and *KpDAS* (D) of strains KpM1, KpM2, KpT1, and KpT2. Total RNA was prepared from the cells of the strains cultured in YNB media containing 0% (white bars), 0.01% (light gray bars), 0.1% (dark gray bars), and 1% (black bars) methanol for 3 h. Transcript levels of *KpAOX1* (C) and *KpDAS* (D) in each strain were analyzed by qRT-PCR. The transcript levels were normalized using *KpACT1* as the reference standard. Relative transcript levels are shown compared to that of the glucose-cultured sample in strain KpM1. Error bars represent standard error values from three independent experiments.

Under methanol-culture conditions, strains KpM1, KpM2, and KpT2 grew normally, but strain KpT1 exhibited slower growth compared to the other strains (Fig. 6A). This suggests that single-copy expression of *KpMIT1* under the *KpTDH3* promoter is not sufficient for optimal growth with methanol. In contrast to the result observed in *C. boidinii*, overexpression of *KpMIT1* did not affect the growth with methanol.

Formaldehyde concentrations in the culture supernatant of strains KpM2 and KpT2 were higher than those of strain KpM1, but decreased rapidly (Fig. 6B). On the other hand, consumption of formaldehyde was delayed in strain KpT1, which likely contributed to its slower growth. The transcript levels of methanol-induced genes *AOX1* and *DAS* in strain KpM2 were higher than those in strain KpM1 across all methanol concentrations, but they showed the CRMI-regulated pattern (Fig. 6C and D). In strain KpT2, although the transcript levels of *AOX1* and *DAS* did not show the CRMI-regulated pattern, they were sufficient for optimal growth with methanol. However, in the strain KpT1, which exhibited retarded growth with methanol, transcript levels of *AOX1* and *DAS* were significantly lower (Fig. 6C and D). These results indicate that the CRMI-dependent expression of *KpMIT1* plays crucial roles in CRMI of methanol-induced genes and that a certain level of KpMit1 is necessary for proper methanol metabolism in *K. phaffii*.

### Coordinated expression of *AOD1* and *DAS1* is important for optimal growth with methanol

**Fig 7:**
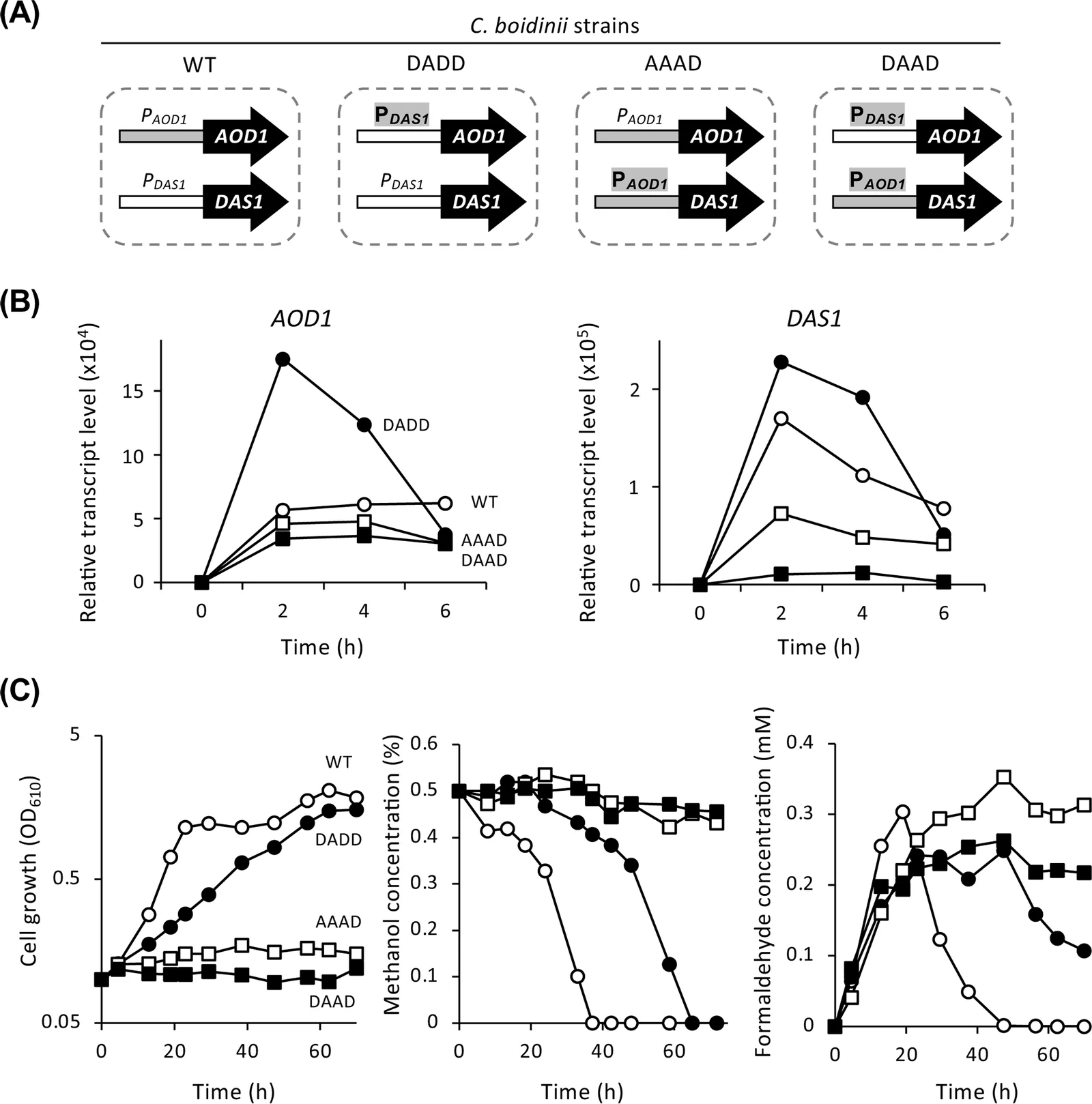
Comparison of promoter activities and their role for methanol metabolism in *C. boidinii*. (A) The model of the promoter swapped strains. See main text for details. (B) Temporal changes of *AOD1* and *DAS1* transcript levels in strains wild-type (WT, open circles), DADD (filled circles), AAAD (open squares), and DAAD (filled squares). Total mRNA was prepared from the cells of each strain cultured in YNB medium containing 0.5% methanol for 2 h. The transcript levels were normalized using *CbACT1* as the standard. Relative transcript levels are shown compared to that of the glucose-cultured sample in WT. Error bars represent standard error values from three independent experiments. (C) Cell growth, methanol consumption, and formaldehyde generation under methanol-culture condition in strains WT (open circles), DADD (filled circles), AAAD (open squares), and DAAD (filled squares). Cells were cultured on YNB medium containing 0.5% methanol.

Expression levels and required time for induction of methanol-induced genes in *C. boidinii* are different among the methanol-inducible promoters (16, 18). We hypothesized that the expression level of *CbMPP1* regulated by CRMI determines the maintenance of transcript levels of multiple methanol-induced genes (e.g., *AOD1*, *DAS1*, *FLD1*) at proper levels for achieving optimal methanol metabolism. To confirm the importance of such coordinated regulation of multiple methanol-induced genes, we investigated the effect of promoter swapping between *AOD1* and *DAS1* genes on the growth with methanol. As shown in Fig. 7A, three kinds of recombinant strains were constructed. Strain DADD expresses both *AOD1* and *DAS1* under the control of *P_DAS1_*, strain AAAD expresses both *AOD1* and *DAS1* under *P_AOD1_*, and strain DAAD expresses *AOD1* under *P_DAS1_* and *DAS1* under *P_AOD1_*.

The transcript levels of *AOD1* and *DAS1* in the wild-type and promoter-replaced strains were quantified under methanol-culture condition. In the wild-type strain, the *DAS1* transcript level rapidly increased after 2 h induction and then gradually decreased, whereas the *AOD1* transcript level was maintained after 2 h induction at a lower level than *DAS1* (Fig. 7B). These results indicated that *P_DAS1_* is stronger and more rapidly induced than *P_AOD1_*. Strain DADD exhibited a significant increase in *AOD1* transcript level due to the strong *P_DAS1_*, and strains AAAD and DAAD showed almost the same level of *AOD1* transcript level as the wild-type strain (Fig. 7B left panel). Strain DADD exhibited almost the same level of *DAS1* transcript as the wild-type strain, whereas strains AAAD and DAAD showed decreased transcript levels (Fig. 7B, right panel). These results demonstrated that the replacement of promoters was performed correctly.

Next, cell growth, methanol consumption, and formaldehyde concentration of the wild-type strain and strains DADD, AAAD, and DAAD were compared under 0.5% methanol-culture condition (Fig. 7C). The growth of strain DADD was slower than that of the wild-type strain, and the methanol consumption rate was also slower than that of the wild-type strain. Formaldehyde concentration was higher at 20 h in the wild-type strain and immediately declined at an early logarithmic growth phase, whereas formaldehyde accumulated for a longer time in strain DADD (Fig. 7C, left panel). Strains AAAD and DAAD showed remarkable defects in cell growth and methanol consumption, even though formaldehyde accumulated at a certain level in these strains (Fig. 7C). These results indicate that the balance of formaldehyde generation and consumption is strictly regulated by the strength and timing of activation of two representative methanol-inducible promoters *P_AOD1_* and *P_DAS1_*.

## DISCUSSION

Methanol-induced gene expression is a distinctive feature of methylotrophic yeasts and is widely used for heterologous gene expression. To achieve efficient protein production, it is important to achieve a balanced C1 metabolism, and it is imperative to elucidate the molecular mechanism of how methanol-induced gene expression is regulated for this purpose. Methylotrophic yeasts have many strategies to minimize the toxicity of formaldehyde, which is a key intermediate of methanol metabolism. Among the strategies, the regulation of multiple methanol-induced genes that encode formaldehyde-generating enzyme AOD and formaldehyde-consuming enzymes, DAS and FLD, is considered to be the most important. So far, many transcriptional regulators involved in the regulation of methanol-induced genes have been identified, and their functions have been elucidated (12, 27). However, it has been unclear how these transcription factors regulate the expression of multiple methanol-induced genes at different levels and the timing of their expression during growth with methanol. In this study, we focused on the transcription factor CbMpp1, which is a homolog of OpMpp1 and KpMit1 and whose expression is induced by methanol, and investigated the regulation of *CbMPP1* expression and its physiological significance for the balanced C1 metabolism under methanol-culture condition.

**Fig 8:**
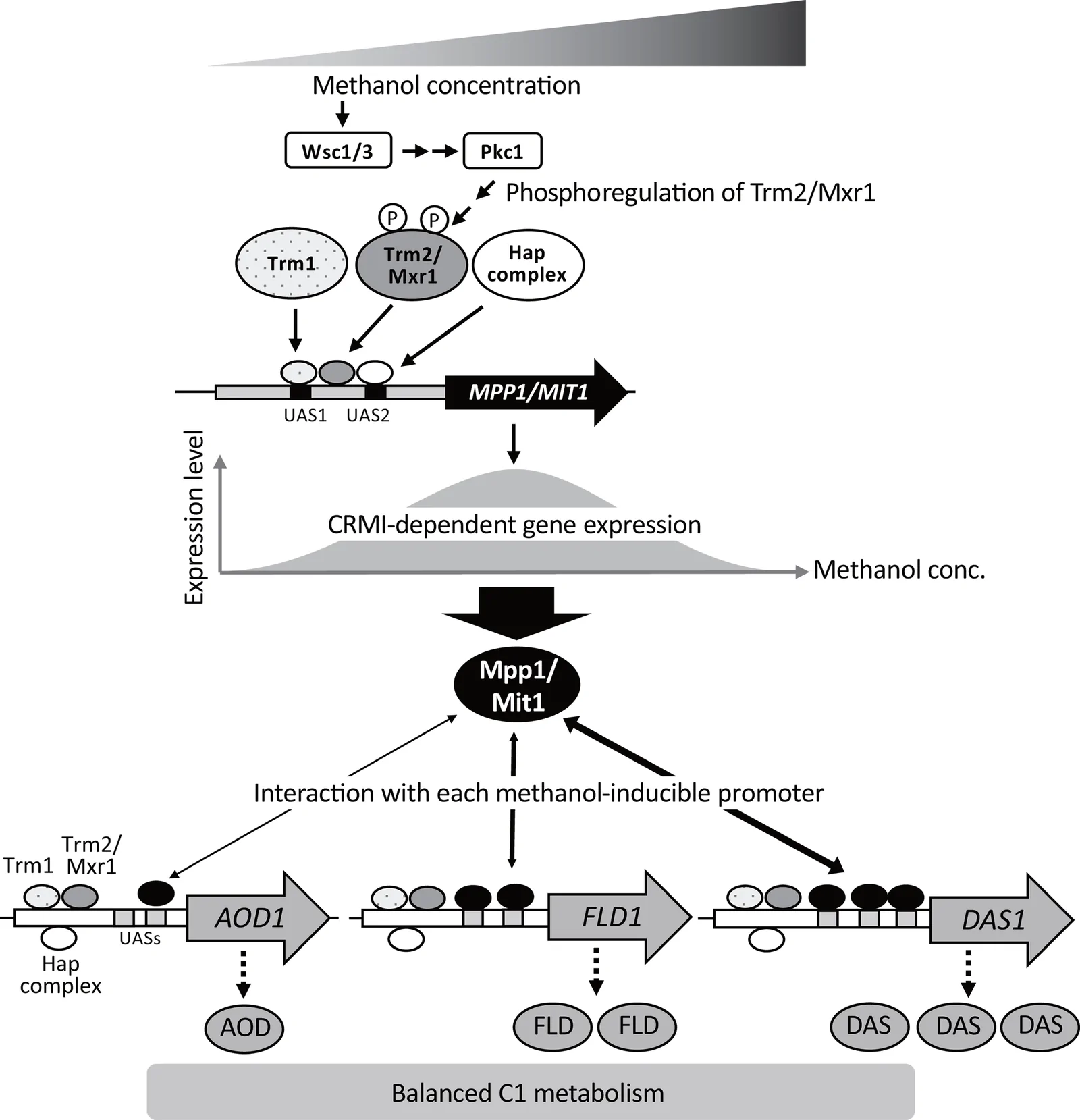
Proposed molecular mechanism for the coordinated regulation of multiple methanol-induced genes mediated by Mpp1/Mit1 in methylotrophic yeasts. Methylotrophic yeasts sense methanol concentration by Wsc1/3 and transmit the signal via Pkc1 to the phosphorylation status of Trm2/Mxr1. The expression of *MPP1/MIT1* is induced under the control of CRMI by transcription factors Trm1, Trm2/Mxr1, and Hap complex. The Mpp1/Mit1 protein level is proposed to control the expression level of each methanol-induced gene (*AOD1, DAS1, FLD1*, etc.) via the binding affinity between Mpp1/Mit1 and each of the promoters, resulting in a balanced C1 metabolism. The width of double-headed arrows represents the strength of binding affinity with each promoter. The interaction may be determined by the affinity and the number of UAS(s) on each promoter.

Our results show that the expression of *CbMPP1* is regulated by CRMI (Fig. 1B), and that lower or higher protein levels of CbMpp1 in the cells lead to the growth delay in methanol medium because of abnormal formaldehyde accumulation (Fig. 3B and C). Furthermore, CRMI of *AOD1*, *DAS1,* and *FLD1* genes was not observed, and their expression levels decreased in strains overexpressing or constitutively expressing *CbMPP1* (Fig. 4 and 5). These results suggest that CbMpp1 expressed in the CRMI-dependent manner regulates the expression of multiple methanol-induced genes at proper expression levels (Fig. 8). In *K. phaffii*, the CRMI-dependent expression of *KpMIT1* is also crucial for proper regulation of methanol-induced genes (Fig. 6). Unlike in *C. boidinii*, overexpression of KpMit1 resulted in higher levels of *AOX1* and *DAS* expression but did not impact growth with methanol.

The importance of proper regulation of multiple methanol-induced genes for the balanced C1 metabolism was supported by the promoter swapping analysis between *AOD1* and *DAS1* (Fig. 7). When *AOD1* was expressed at the same level and times as *DAS1*, the cells were able to cope with the formaldehyde accumulation and could grow on methanol. However, when *DAS1* was expressed at the same level and times as *AOD1*, formaldehyde accumulated in the medium was not consumed, and the cells could not grow on methanol probably due to formaldehyde toxicity. DAS should be induced strongly by methanol at the early growth phase to immediately consume formaldehyde produced by AOD. Therefore, the rapid and strong expression of *DAS1* is critical for the balanced C1 metabolism and growth with methanol. Thus, the expressions of *AOD1* and *DAS1* are coordinately regulated to maintain the optimum level of formaldehyde for optimal growth with methanol, and this regulation might be achieved by CRMI-regulated CbMpp1.

Based on all the results obtained and reported so far, we propose a model of CRMI-dependent regulation of multiple methanol-induced genes mediated by Mpp1/Mit1 transcription factor in methylotrophic yeasts (Fig. 8). These yeasts sense environmental methanol concentration by Wsc1/3 and transmit the methanol signal to Trm2/Mxr1 via Pkc1 by regulation of phosphorylation status of Trm2/Mxr1 (13, 15). The expression of *MPP1/MIT1* is induced under the control of CRMI by transcription factors Trm1, Trm2/Mxr1, and Hap complex, and the methanol-concentration dependency of the transcription of *MPP1/MIT1* is regulated in a Trm2/Mxr1-dependent manner. The expression level of *MPP1/MIT1* and the binding affinity of Mpp1/Mit1 to each methanol-inducible promoter are supposed to regulate the fine-tuned gene expression of other multiple methanol-induced genes at different expression levels. We propose that Mpp1/Mit1 protein level controls the expression level of each methanol-induced gene (*AOD1, DAS1, FLD1*, etc.) via binding affinity between Mpp1/Mit1 and each of the promoters, resulting in a balanced C1 metabolism to minimize the toxicity of formaldehyde. Since KpMxr1 was shown to bind multiple regions in the *AOX1* promoter in *K. phaffii* (22), it is possible that Mpp1/Mit1 binds to multiple UASs in each methanol-induced promoter. During evolution, the affinity of Mpp1/Mit1 and UASs of each promoter may have been optimized for balanced gene expression of methanol-induced genes and C1 metabolism. Further studies on the molecular action of transcription factors, for example, determining the binding affinity between UASs of each promoter and Mpp1/Mit1 protein, will facilitate further understanding of the regulatory mechanism on gene expression in methylotrophic yeasts and its application in promoting production of heterologous proteins.

## MATERIALS AND METHODS

### Strains, media, and culture conditions

*C. boidinii* and *K. phaffii* strains and plasmids used in this study are shown in Tables S1 and S2, respectively. The yeast cells were grown at 28°C on YPD (1% yeast extract, 2% peptone, 2% glucose) or YNB medium (0.67% yeast nitrogen base without amino acids, pH 6.0) with gentle shaking (120 rpm). Two percent glucose, 0.5% ethanol, or several concentrations of methanol were used as carbon sources in YNB medium. All components other than the carbon sources used in these media were purchased from Difco Becton Dickinson and Company (Franklin Lakes, NJ). Yeast growth was monitored by measuring the optical density (OD) at 610 nm. *Escherichia coli* cells were grown in LB medium (1% tryptone, 0.5% yeast extract, 0.5% NaCl), supplemented with ampicillin (50 mg/L) at 37°C when required.

### Plasmid construction and gene disruption

The plasmids and oligonucleotide primers used in this study are shown in Tables S3 and S4, respectively. The procedure for construction of the plasmids used in this study is described in Supplementary Materials and Methods.

The 6.3 kbp fragment was amplified by PCR with the primer pair MPP1up-PstI-Fw/MPP1down-PstI-Rv using the *CbMPP1* disruption vector pMPP1D as a template. The fragment was introduced into *C. boidinii* TK62-*ura3* using the Fast Yeast Transformation Kit (GE Healthcare, IL, USA) to obtain the *Cbmpp1*Δ strain. In addition, the *Cbmpp1*Δ strain was converted to uracil auxotrophy by 5-fluoroorotidic acid selection, yielding the *Cbmpp1*Δ*ura3* strain. The 3.0 kbp fragment was amplified by PCR with the primer pair Kpmit1d-Fw/Kpmit1d-Rv using pKI003 (13) as a template. The fragment was used to transform *K. phaffii* PPY12 by electroporation, yielding the *Kpmit1*Δ strain. Proper gene disruptions were confirmed by colony PCR.

### RNA isolation and qRT-PCR

Extraction of total RNA and cDNA synthesis were performed as previously described (28). qRT-PCR was performed with SYBR Premix Ex Taq (Takara Bio) using the Light Cycler Instrument (Roche Diagnostics, Basel, Switzerland) or QuantStudio 1 Real-Time PCR System (Thermofisher Scientific, Waltham, MA). The primers used in qRT-PCR are listed in Table S3. Transcript levels of all genes in methanol culture were normalized to *CbACT1* or *KpACT1.*

### Construction of reporter strains and APase activity assay

The construction of the plasmids used in promoter-reporter analyses is described in Supplementary Materials and Methods. The linearized plasmids were transformed into *C. boidinii* TK62-*ura3* by chromosome integration at the *URA3* locus, and single-copy transformants were isolated using Southern blot analysis as described in Supplementary Materials and Methods. Yeast cells were grown in YPD and YNB medium containing 2% glucose and 0.5% yeast extract and then transferred to 0.7% methanol medium for 8 h at a cell density OD_610_ of 1.0. Cells were harvested, washed twice with 50 mM acetate buffer (pH 4.0), and suspended in 200 µL of 50 mM acetate buffer (pH 4.0) with the appropriate dilution of the cells. APase activity was measured as described previously (18, 29).

### Chromatin immunoprecipitation

Cultured cells equivalent to 100 OD_610_ units were harvested and treated with 1% formaldehyde for 10 min. Also, 125 mM glycine solution was added, and the cells were incubated at room temperature for 5 min, collected after centrifugation (2,000 *g*, 5 min, 4°C), and were resuspended in lysis buffer (50 mM HEPES-KOH, 140 mM NaCl, 1 mM EDTA, 1% Triton X-100, 0.1% sodium deoxycholate, proteinase K [Invitrogen], pH 7.5). Subsequently, the cells were broken by glass beads (φ 0.5 mm), and the fixed chromatin was broken by sonication using a Bioruptor (Diagenode). MAGnify Chromatin Immunoprecipitation System (Invitrogen) was used according to the product manual for immunoprecipitation, which was performed using an anti-GFP polyclonal antibody (Molecular Probes, Eugene, OR) at the recommended dilution. The primers used for the amplification of the target DNA regions are listed in Table S4.

### Fluorescence microscopy observation

Cultured cells were fixed with 70% ethanol for 30 min and subsequently incubated in 4′,6-diamidino-2-phenylindole (DAPI) solution (50 µg/L, Nacalai tesque) for 20 min. Observations were carried out with an IX81 fluorescence microscope (Olympus, Tokyo, Japan). Fluorescent images were captured with a charge-coupled device camera (SenSys; PhotoMetrics, Tucson, AZ) at the fixed exposure time of 200 ms in DIC field, 1,000 ms in YFP field, and 50 ms in DAPI field using MetaMorph software (Universal Imaging, West Chester, PA).

### Determination of methanol and formaldehyde concentration

To determine methanol and formaldehyde concentration, cultured media were harvested at the same indicated time points as for cell growth measurement and centrifuged at 6,000 *g* for 2 min at 4°C. Methanol concentration in the culture supernatant was determined as described previously using a GC-2014 (Shimadzu Co, Kyoto, Japan) gas chromatograph (30). Formaldehyde concentration in the culture supernatant was monitored using a previously described method (31). The samples were diluted 1:1 to Nash reagent (2 M ammonium acetate, 20 mM acetyl acetone, and 50 mM acetic acid). Formaldehyde solutions (0.04 mM–0.2 mM) were used for calibration. After incubation for 30 min at 30°C, the absorbance at 410 nm of each sample was measured using the microplate spectrophotometer (Tecan, Sunrise Rainbow Thermo RC-R, F039300RTRCR, Männedorf, Switzerland).

### Statistical analysis

All data were obtained from three independent biological replicates and presented as means ± SE. Student’s *t*-test was performed to determine the differences among grouped data. Statistical significance was assessed at *P* < 0.05. For comparison between some groups, a parametric one-way analysis of variance based on the Tukey-Kramer test was used.

## Data Availability

The nucleotide sequences of *CbMPP1* and *CbTDH3* have been submitted to DDBJ and assigned accession numbers, LC764437 and LC764438, respectively. The raw dataset of qRT-PCR analysis, promoter activity measurements, and formaldehyde quantitation are available in the Kyoto University Research Information repository, https://doi.org/10.14989/291922.

## SUPPLEMENTAL MATERIAL

Supplemental material spectrum.02853-24-s0001.pdf Supplemental materials and methods, Tables S1 to S4, and Figures S1 to S5.

ASM does not own the copyrights to Supplemental Material that may be linked to, or accessed through, an article. The authors have granted ASM a non-exclusive, world-wide license to publish the Supplemental Material files. Please contact the corresponding author directly for reuse.
