# Supplementary material for: Methanol-induced transcription factor Mpp1 regulates the coordinated expression of multiple genes to achieve a balanced C1 metabolism in the methylotrophic yeast Candida boidinii
Source: Microbiol Spectr. 2025 Mar 31;13(5):e02853-24. doi: 10.1128/spectrum.02853-24 (PMC12054124; doi:10.1128/spectrum.02853-24)
Supplement: Supplemental material — s and methods, Tables S1 to S4, and Figures S1 to S5. [file spectrum.02853-24-s0001.pdf]

Supplementary Material

Methanol-induced transcription factor Mpp1 regulates the coordinated expression of multiple genes to achieve a balanced C1 metabolism in the methylotrophic yeast *Candida boidinii*

Koichi Inoue, Nono Saso, Kosuke Iwase, Zhenyu Zhai, Takahito Tsuji, Kazuyoshi Tabata, Rie Sano, Hiroya Yurimoto and Yasuyoshi Sakai

Division of Applied Life Sciences, Graduate School of Agriculture, Kyoto University, Kitashirakawa-Oiwake, Sakyo-ku, Kyoto 606-8502, Japan.

*Correspondence: Hiroya Yurimoto, Ph.D.
Division of Applied Life Sciences, Graduate School of Agriculture, Kyoto University, Kitashirakawa-Oiwake, Sakyo-ku, Kyoto 606-8502, Japan.
Tel.: +81 75 753 6385; fax: +81 75 753 6454;
e-mail: yurimoto.hiroya.5m@kyoto-u.ac.jp

This file includes:
Supplementary Materials and Methods
Tables S1 to S4
Figures S1 to S5
References

Supplementary Materials and Methods

Plasmid construction.

A deletion cassette for the *CbMPP1* gene was constructed to delete almost all of the open reading frame region as follows: the primer pairs MPP1up-PstI-Fw/ MPP1up-NotI-XhoI-Rv and MPP1down-NotI-XhoI-Fw/ MPP1down-PstI-Rv were used to amplify 1.7-kbp (MPP1-Up) and 0.7-kbp (MPP1-Down) fragments using the genomic DNA as a template. These fragments were connected and amplified by PCR with the primers MPP1up-PstI-Fw/ MPP1down-PstI-Rv. The fragment was inserted into pMD20 (Takara Bio, Otsu, Japan), yielding the plasmid pMD20MPP1. The 5.1-kbp NotI-XhoI digested fragment of pMD20MPP1 and the 4.6-kbp NotI-XhoI digested fragment from pSPR harboring *C. boidinii URA3* gene (1) were ligated, yielding the *CbMPP1* disruption vector pMPP1D.

The empty vector pCBU1 possessing *URA3* marker gene was prepared from pAPU1 (2) by In-Fusion HD Cloning Kit (Takara Bio) using the primer pair Inverse-tAOD1-Fw/ Infusion-pCBU1-Rv. The *CbMPP1* coding region and its promoter were amplified by PCR with the primer pair Pmpp1-SacI-Fw/ MPP1ORF-PstI-Rv from *C. boidinii* genomic DNA as a template. The YFP-encoding region was amplified by PCR with the primer pair PstI-YFP-Fw/ NheI-YFP-Rv using pTYT as the template (3). These fragments were digested by SacI, PstI and NheI and then ligated with pCBU1, resulting in pMMY1.

The *CbMPP1* promoter region was amplified by PCR with the primer pair Pmpp1-SacI-Fw/ Pmpp1-BamHI-Rv from pMMY1, and the DNA fragment of *S. cerevisiae PHO5* gene encoding acid phosphatase was prepared by BamHI and SpeI digestion from pAPU1 (2). They were ligated with pCBU1, yielding pMPU1.

The DNA region from –1400 to –1 in *CbMPP1* promoter was amplified from pMPU1 using the primer pairs Infusion-Pmpp1-1400-Fw/ Infusion-Pmpp1-1-Rv. pMPU1 was digested by BamHI and SacI and used as a vector DNA. These DNA fragments were ligated by In-Fusion HD Cloning Kit (Takara Bio), yielding pMPUΔ1700-1400. In the same way, pMPUΔ1700-1300, pMPUΔ1700-1200, pMPUΔ1700-1000 and pMPUΔ1700-900 were prepared using the primer pairs Infusion-Pmpp1-1300-Fw/ Infusion-Pmpp1-1-Rv, Infusion-Pmpp1-1200-Fw/ Infusion-Pmpp1-1-Rv, Infusion-Pmpp1-1000-Fw/ Infusion-Pmpp1-1-Rv and Infusion-Pmpp1-900-Fw/ Infusion-Pmpp1-1-Rv, respectively.

The DNA region from –800 to –1 in *CbMPP1* promoter was amplified from pMPU1 using the primer pairs NEB-Pmpp1-800-Fw/ NEB-Pmpp1-1-Rv. And the region from –1700 to –1000, –1700 to –1100, –1700 to –1200, –1700 to –1300 and –1700 to –1400 were amplified using the primer pairs NEB-Pmpp1-1700-Fw/ NEB-Pmpp1-1000-Rv, NEB-Pmpp1-1700-Fw/ NEB-Pmpp1-1100-Rv, NEB-Pmpp1-1700-Fw/ NEB-Pmpp1-1200-Rv, NEB-Pmpp1-1700-Fw/ NEB-Pmpp1-1300-Rv and NEB-Pmpp1-1700-Fw/ NEB-Pmpp1-1400-Rv, respectively. pMPU1 was digested by BamHI and SacI and used as a vector DNA. DNA fragments of –800 to –1, –1700 to –1000 and the vector were ligated to obtain pMPUΔ1000-800. pMPUΔ1100-800, pMPUΔ1200-800, pMPUΔ1300-800 and pMPUΔ1400-800 were prepared in the same method.

pMPUΔ1400-1350 was prepared from the fragment amplified by the primer pair NEB-d1400-1350-Fw/ NEB-d1400-1350-Rv. pMPUΔ1400-1250, pMPUΔ1200-1100, pMPUΔ1100-1000 and pMPUΔ1000-900 were created in the same way using the primer pairs NEB-d1400-1250-Fw/ NEB-d1400-1250-Rv, NEB-d1200-1100-Fw/ NEB-d1200-1100-Rv, NEB-d1100-1000-Fw/ NEB-d1100-1000-Rv and NEB-d1000-900-Fw/

NEB-d1000-900-Rv, respectively.

The DNA region from –525 to –1 in *CbACT1* promoter was amplified from pACT1 using the primer pair NEB-Pact1-ScPHO5-Fw/ NEB-Pact1-ScPHO5-Rv. pMPU1 was digested by BamHI and SacI, and used as a vector DNA. These fragments were ligated by NEBuilder HiFi DNA Assembly (New England Biolabs, Ipswich, MA, USA) and then obtain pACTPU1. I defined the DNA region from –1350 to –1250 and –1000 to –900 as "UAS1" and "UAS2", respectively. They were amplified from pMPU1 using the primer pairs NEB-1350-1250-ACT-Fw/ NEB-1350-1250-ACT-Rv and NEB-1000-900-ACT-Fw/ NEB-1000-900-ACT-Rv. Subsequently, they were ligated with the vector DNA amplified from pACTPU1 using primer pairs Inverse-pACTPU1-Fw/ Inverse-pACTPU1-Rv, resulting in pUAS1-ACTPU1 and pUAS2-ACTPU1, respectively.

The promoter region of *CbTDH3* encoding glyceraldehyde-3-phosphate dehydrogenase was amplified by PCR from *C. boidinii* genomic DNA as a template using primer pairs EcoRI-Ptdh3-Fw/ NotI-Ptdh3-Rv. The fragment was ligated with pCBU1 vector digested by EcoRI and BamHI, resulting in pTDH3eI. *CbMPP1* gene and the DNA sequence of fluorescent protein YFP were amplified from pMMY1 using the primer pair NEB-Ptdh3-Mpp1YFP-Fw/ NEB-Ptdh3-Mpp1YFP-Rv. Subsequently, the fragment was ligated with vector DNA amplified from pTDH3eI using the primer pair Inverse-pTDH3eI-Fw/ Inverse-pTDH3eI-Rv by NEBuilder HiFi DNA Assembly (New England Biolabs), resulting in pTMY1.

AOD1 gene was amplified by PCR with the primer pair NotI-AOD1-Fw/ NotI-AOD1-Rv from *C. boidinii* genomic DNA as a template. The 2.0-kbp DNA fragment was digested with NotI and ligated to the NotI site of pNOTdI (4), resulting in pDAU1. *DAS1* gene was amplified with the primer pair NotI-DAS1-Fw/ NotI-DAS1-Rv and

ligated to the NotI site of pNOTeI (5) or pNOTeLI (6) in the same way, yielding pADU1 or pADL1 respectively.

The *KpMIT1* coding region and its promoter were amplified by PCR with the primer pair Infusion-KpnI-Pmit1-pIB1-Fw/ Infusion-BamHI-MIT1-pIB1-Rv from *K. phaffii* genomic DNA as a template. pSN303 (7) was digested by KpnI and BamHI and used as a vector DNA. These DNA fragments were ligated by In-Fusion HD Cloning Kit (Takara Bio), yielding pMMF. The promoter region of *KpTDH3* (PAS_chr2-1_0437) encoding glyceraldehyde-3-phosphate dehydrogenase was amplified by PCR from *K. phaffii* genomic DNA as a template using primer pairs Inf-Pgap-MIT1-Fw/ Inf-Pgap-MIT1-Rv. Subsequently, the fragment was ligated with vector DNA amplified from pMMF using the primer pair Inf-MIT1-pIB1arg-Fw/ Inf-KpnI-pIB1arg-Rv by In-Fusion HD Cloning Kit, yielding pTMFA. MIT1 coding gene and FLAG tag region were amplified by PCR with the primer pair Inf-MIT1FLAG-Fw/ Inf-MIT1FLAG-Rv from pTMFA. Subsequently, the fragment was ligated with vector DNA amplified from pIB1 (8) using the primer pair Inf-pIB1his-Fw/ Inf-pIB1his-Rv by In-Fusion HD Cloning Kit, yielding pTMFH.

Southern blot analysis.

The DNA probe for confirming transformation and plasmid copy number was designed for the *URA3* gene. A DNA fragment was prepared by PCR from genomic DNA using primer pair Southern-URA3-Fw/ Southern-URA3-Rv. The purified DNA fragment was denatured by boiling for 5 min and then incubated on ice for 5 min. 10 mg/L of the fragment was labeled with alkaline phosphatase (Amersham Gene Images AlkPhos Direct Labelling and Detection System, GE Healthcare). The genomic DNA was

digested with HindIII and electrophoresed on a 1.0% agarose gel. Blotting and detection were performed as described previously (9).

Protein extraction and Immunoblot analysis.

The cultured cells equivalent to 2 OD_{610} units were collected for protein extraction. They were suspended in 0.2 N NaOH solution containing 0.5% β-mercaptoethanol for 10 min on ice and trichloroacetic acid was added to a final concentration of 10% v/v for cell lysis. The samples were centrifuged (20,000 g, 5 min, 4°C) and protein pellets were washed three times with 100% acetone by brief sonication. Subsequently, protein pellets were resuspended in the sample buffer (62.5 mM Tris-HCl, 2% SDS, 10% Glycerol, 5% β-mercaptoethanol, 0.005% BPB, pH 6.8) and incubated at 65 °C for 10 min.

The samples were first centrifuged at 20,000 g for 1 min. 10 μL of the supernatant was electrophoresed on 7-10% acrylamide SDS-PAGE gel. Precision Plus Protein Dual Color Standard (Bio-Rad, Hercules, USA) was used as a protein-loading marker. The proteins were transferred to an Immobilon-P PVDF membrane (0.2 μm, Merck KGaA, Darmstadt, Germany) by semidry blotting (Bio-Rad). The membranes were incubated in Blocking One (Nakalai tesque, Kyoto, Japan) and then in the solution containing anti-YFP antibody (anti-GFP antibody, JL-8, mouse monoclonal, Takara Bio), anti-AOD antibody (rabbit polyclonal; (10), anti-DAS antibody (rabbit polyclonal; (10) or anti-β-actin (mAbcam 8224, mouse monoclonal, Abcam, Cambridge, UK) at dilutions recommended in the protocol with TBS-T buffer (50 mM Tris-HCl, 138 mM NaCl, 2.7 mM KCl, 0.05% Tween 20, pH 7.5). The membranes were washed three times with TBS-T buffer and incubated with anti-mouse-HRP (No. 330, goat polyclonal, Merck Millipore, Darmstadt, Germany) or anti-rabbit-HRP (#7074, goat polyclonal, Abcam,

Cambridge, UK) at a 1:5,000 dilution for 1 h. Finally, bound secondary antibodies were detected using Western Lightning (Perkin-Elmer Life Science, Waltham, MA) and the signals were detected using Lummino-Graph II (ATTO, Tokyo, Japan).

TABLE S1 *C. boidinii* strains used in this study

Designation	Genotype	Reference
AOU1	Wild type	(11)
TK62	*ura3*	(12)
Cbtrm1Δura3	*Cbtrm1Δ*, *ura3*	(3)
Cbtrm2Δura3	*Cbtrm2Δ*, *ura3*	(13)
Cbtrm1ΔCbtrm2Δura3	*Cbtrm1ΔCbtrm2Δ*, *ura3*	(13)
Cbhap3Δura3	*Cbhap3Δ*, *ura3*	(14)
Cbmpp1Δ	*Cbmpp1Δ::(pMPP1D)*, *URA3*	This study
Cbmpp1Δura3	*Cbmpp1Δ*, *ura3*	This study
PHO-FL	TK62, *ura3*::(pMPU1)	This study
PHOtrm1Δ-FL	*Cbtrm1Δura3*, *ura3*::(pMPU1)	This study
PHOtrm2Δ-FL	*Cbtrm2Δura3*, *ura3*::(pMPU1)	This study
PHOtrm1Δtrm2Δ-FL	*Cbtrm1Δtrm2Δura3*, *ura3*::(pMPU1)	This study
PHOhap3Δ-FL	*Cbhap3Δura3*, *ura3*::(pMPU1)	This study
PHOmpp1Δ-FL	*Cbmpp1Δura3*, *ura3*::(pMPU1)	This study
PHOΔ1700-1400	TK62, *ura3*::(pMPUΔ1700-1400)	This study
PHOΔ1700-1300	TK62, *ura3*::(pMPUΔ1700-1300)	This study
PHOΔ1700-1200	TK62, *ura3*::(pMPUΔ1700-1200)	This study
PHOΔ1700-1000	TK62, *ura3*::(pMPUΔ1700-1000)	This study
PHOΔ1700-900	TK62, *ura3*::(pMPUΔ1700-900)	This study
PHOΔ1000-800	TK62, *ura3*::(pMPUΔ1000-800)	This study
PHOΔ1100-800	TK62, *ura3*::(pMPUΔ1100-800)	This study
PHOΔ1200-800	TK62, *ura3*::(pMPUΔ1200-800)	This study
PHOΔ1300-800	TK62, *ura3*::(pMPUΔ1300-800)	This study
PHOΔ1400-800	TK62, *ura3*::(pMPUΔ1400-800)	This study
PHOΔ1400-1350	TK62, *ura3*::(pMPUΔ1400-1350)	This study
PHOΔ1400-1250	TK62, *ura3*::(pMPUΔ1400-1250)	This study
PHOΔ1200-1100	TK62, *ura3*::(pMPUΔ1200-1100)	This study
PHOΔ1100-1000	TK62, *ura3*::(pMPUΔ1100-1000)	This study
PHOΔ1000-900	TK62, *ura3*::(pMPUΔ1000-900)	This study
PHOΔ2Δ1400-1250	*Cbtrm2Δura3*, *ura3*::(pMPUΔ1400-1250)	This study
PHOΔ2Δ1000-900	*Cbtrm2Δura3*, *ura3*::(pMPUΔ1000-900)	This study
PHO-A	TK62, *ura3*::(pACTPU1)	This study

TABLE S1 Continued

Designation	Genotype	Reference
PHO-1A	TK62, *ura3*::(pUAS1-ACTPU1)	This study
PHO-2A	TK62, *ura3*::(pUAS2-ACTPU1)	This study
M1	*Cbmpp1Δura3*, *ura3*::(pMMY1)	This study
M2	TK62, *ura3*::(pMMY1)	This study
T1	*Cbmpp1Δura3*, *ura3*::(pTMY1)	This study
T3	*Cbmpp1Δura3*, *ura3*::(pTMY1; 3 copies)	This study
aod1Δura3	TK62, *aod1Δ, ura3*	(15)
das1Δura3	TK62, *das1Δ, ura3*	(16)
aod1Δdas1Δura3leu2	TK62, *aod1Δdas1Δ, ura3, leu2*	This study
DADD	*aod1Δura3, ura3*::(pDAU1)	This study
AAAD	*das1Δura3, ura3*::(pADU1)	This study
DAAD	*aod1Δdas1Δura3leu2, ura3*::(pDAU1), *leu2*::(pADL1)	This study

TABLE S2 *K. phaffii* strains used in this study

Designation	Genotype	Reference
PPY12	*arg4, his4*	(17)
Kpmit1Δ	PPY12, *mit1Δ*::Bsd^{R}, *arg4, his4*	This study
KpM1	*Kpmit1Δ, arg4*::(pMMF), *his4*::(pIB1)	This study
KpM2	PPY12*, arg4*::(pMMF) , *his4*::(pIB1)	This study
KpT1	*Kpmit1Δ, arg4*::(pTMFA) , *his4*::(pIB1)	This study
KpT2	*Kpmit1Δ, arg4*::(pTMFA), *his4*::(pTMFH)	This study

TABLE S3 Plasmids used in this study

Designation	Description	Reference
pAPU1	P_{AOD1}-*ScPHO5*, *URA3*	(2)
pCBU1	*URA3*	This study
pMPP1D	*Cbmpp1Δ::URA3*	This study
pMPU1	P_{CbMPP1}-*ScPHO5*, URA3	This study
pMPUΔ1700-1400	pMPU1/ P_{CbMPP1} [from -1400 to -1]-*ScPHO5*	This study
pMPUΔ1700-1300	pMPU1/ P_{CbMPP1} [from -1300 to -1]-*ScPHO5*	This study
pMPUΔ1700-1200	pMPU1/ P_{CbMPP1} [from -1200 to -1]-*ScPHO5*	This study
pMPUΔ1700-1000	pMPU1/ P_{CbMPP1} [from -1000 to -1]-*ScPHO5*	This study
pMPUΔ1700-900	pMPU1/ P_{CbMPP1} [from -900 to -1]-*ScPHO5*	This study
pMPUΔ1400-1350	pMPU1/ P_{CbMPP1} [from -1700 to -1400 and 1350 to -1]-*ScPHO5*	This study
pMPUΔ1400-1250	pMPU1/ P_{CbMPP1} [from -1700 to -1400 and 1250 to -1]-*ScPHO5*	This study
pMPUΔ1200-1100	pMPU1/ P_{CbMPP1} [from -1700 to -1200 and 1100 to -1]-*ScPHO5*	This study
pMPUΔ1100-1000	pMPU1/ P_{CbMPP1} [from -1700 to -1100 and 1000 to -1]-*ScPHO5*	This study
pMPUΔ1000-900	pMPU1/ P_{CbMPP1} [from -1700 to -1000 and 900 to -1]-*ScPHO5*	This study
pACT1	P_{ACT1}, *URA3*	(18)
pACTPU1	pMPU1/ P_{ACT1}-*ScPHO5*	This study
pUAS1-ACTPU1	pMPU1/ P_{CbMPP1} [from -1350 to -1250]-P_{ACT1}-*ScPHO5*	This study
pUAS2-ACTPU1	pMPU1/ P_{CbMPP1} [from -1000 to -900]-P_{ACT1}-*ScPHO5*	This study
pMMY1	pCBU1/ P_{CbMPP1}-*CbMPP1*-YFP	This study
pTMY1	pCBU1/ P_{TDH3}-*CbMPP1*-YFP	This study
pNOTeI	P_{AOD1}, *URA3*	(5)
pNOTdI	P_{DAS1}, *URA3*	(4)
pNOTeLI	P_{AOD1}, *LEU2*	(6)
pDAU1	P_{DAS1}-*AOD1*, *URA3*	This study
pADU1	P_{AOD1}-*DAS1*, *URA3*	This study
pADL1	P_{AOD1}-*DAS1*, *LEU2*	This study
pMPUΔ1000-800	pMPU1/ P_{CbMPP1} [from -1000 to -800]-*ScPHO5*	This study
pMPUΔ1100-800	pMPU1/ P_{CbMPP1} [from -1100 to -800]-*ScPHO5*	This study
pMPUΔ1200-800	pMPU1/ P_{CbMPP1} [from -1200 to -800]-*ScPHO5*	This study
pMPUΔ1300-800	pMPU1/ P_{CbMPP1} [from -1300 to -800]-*ScPHO5*	This study
pMPUΔ1400-800	pMPU1/ P_{CbMPP1} [from -1400 to -800]-*ScPHO5*	This study

TABLE S3 Continued

Designation	Description	Reference
pIB1	*KpHIS4*	(19)
pNT204	*KpARG4*	(20)
pSN303	P_{KpMXR1}-*MXR1*-5xFLAG/pNT204	(8)
pMMF	P_{KpMIT1}-*MIT1*-5xFLAG/pNT204	This study
pTMFA	P_{KpTDH3}-*MIT1*-5xFLAG/pNT204	This study
pTMFH	P_{KpTDH3}-*MIT1*-5xFLAG/pIB1	This study
pKI003	*Kpmit1Δ*::Bsd^R	(7)

TABLE S4 Oligonucleotide primers used in this study

Designation	DNA sequence (5'-3')
Inverse-tAOD1-Fw	GCGGCCGCTAATTCAACAAG
Infusion-pCBU1-Rv	TGAATTAGCGGCCGCGAATTCGTAATCATGGTCATAGCTGTTTCC
MPP1up-PstI-Fw	AACTGCAGCGCTTGTTACGTAATCTTGCTCTTTGTA
MPP1up-NotI-XhoI-Rv	GCGGCCGCATTCTTATCTCGAGCCGTATATGAAATAAATGAAGTAAG
MPP1down-NotI-XhoI-Fw	CGGCTCGAGATAAGAATGCGGCCGCTTAGTTGATTTTTGGAATAGTG
MPP1down-PstI-Rv	AACTGCAGCTAAATTACAAAAGCATCAATTAATATCAC
Pmpp1-SacI-Fw	CGAGCTCCGCTTGTTACGTAATCTTGCTCTTTGTA
MPP1ORF-PstI-Rv	AACTGCAGAGTTTCACTATTCCAAAAATCAACTAACAT
Infusion-Pmpp1-1400-Fw	GGGAACAAAAGCTGGAGCTCTGAATCAAAATAGTTTATGGTC
Infusion-Pmpp1-1-Rv	TCGAATTTGCGGATCCTATATGAAATAAATGAAGTAAGTA
Infusion-Pmpp1-1300-Fw	GGGAACAAAAGCTGGAGCTCGATTGTTTTTTAATTGGATT
Infusion-Pmpp1-1200-Fw	GGGAACAAAAGCTGGAGCTCGATTACGTAATGCACTATTTAAAGTTTCC
Infusion-Pmpp1-1000-Fw	GGGAACAAAAGCTGGAATTAGCAGGCAATTAGCATT
Infusion-Pmpp1-900-Fw	ACAAAAGCTGGAGCTCGTTGAGTTCCTTCTTTGTTTGTTTC
NEB-Pmpp1-800-Fw	TGATCATGTTCTTGTTTATATGTTTATGG
NEB-Pmpp1-1-Rv	AATCTCGAATTTGCGGATCCTATATGAAATAAATGAAGTAAGTAAGAAAGTAAG
NEB-Pmpp1-1700-Fw	ACAAAAGCTGGAGCTCCGCTTGTTACGTAATCTTGC
NEB-Pmpp1-1000-Rv	ACAAGAACATGATCAGCTTTCAATCAACTGAAAGTGAG
NEB-Pmpp1-1100-Rv	ACATATAAACAAGAACATGATCATATAGTAATATATTAACATTTTATAATGTACTGTC
NEB-Pmpp1-1200-Rv	ACAAGAACATGATCACACACAACTCAAATTTTTTTATAGTTTTAAA
NEB-Pmpp1-1300-Rv	ACAAGAACATGATCACGATTTATCCTATTCATATTAAGTATAAATAAC
NEB-Pmpp1-1400-Rv	ACAAGAACATGATCATTGTTATATTTTATTATATTGTACTGTATTGTATTGTATTGT
NEB-d1400-1350-Fw	TAATAAAATATAACATAGTATTATATAGTTATAGTTATTTATACTTAATATGAATAGG
NEB-d1400-1350-Rv	TGTTATATTTTATTATATTGTACTGTATTGTATTGTATTGTATTG
NEB-d1400-1250-Fw	TAATAAAATATAACAACTGTATATTAAATTATATATTTAAAACTATAAAAAAATTTGAG
NEB-d1400-1250-Rv	TGTTATATTTTATTATATTGTACTGTATTGTATTGTATTGTATTG
NEB-d1200-1100-Fw	AGTACATATTGATATATAGTAGTATATGGTAGGAATATTATATTG
NEB-d1200-1100-Rv	ATATCAATATGTACTACACAACTCAAATTTTTTTATAGTTTTAAAT
NEB-d1100-1000-Fw	CAATTAGCAGGCAATTAGCATTTAATTAGCATTTAATTAGC
NEB-d1100-1000-Rv	ATTGCCTGCTAATTGATAGTAATATATTAACATTTTATAATGTACTG
NEB-d1000-900-Fw	CGTTGAGTTCCTTCTTTGTTTGTTTCTGTTTC
NEB-d1000-900-Rv	AGAAGGAACTCAACGCTTTCAATCAACTG
NEB-Pact1-ScPHO5-Fw	ACAAAAGCTGGAGCTCTTAGCCACTTTAACCACTCTGC
NEB-Pact1-ScPHO5-Rv	AATCTCGAATTTGCGGATCCAATATATATTAAATTAAATTTATAAAATCTATCTAGGTTATAAATC TAG
NEB-1350-1250-ACT-Fw	ACAAAAGCTGGAGCTTAGTATTATATAGTTATAGTTATTTATACTTAATATGAATAGC
NEB-1350-1250-ACT-Rv	AAGTGGCTAAGAGCTAAATTAATAAATTAAATTAAAAATTTTTCAATCC
NEB-1000-900-ACT-Fw	ACAAAAGCTGGAGCTAATTAGCAGGCAATTAGC
NEB-1000-900-ACT-Rv	AAGTGGCTAAGAGCTGGAACGGAACTGAACG
Inverse-pACTPU1-Fw	AGCTCTTAGCCACTTTAACCACTCTG
Inverse-pACTPU1-Rv	AGCTCCAGCTTTTGTTCCCTTTAG
PstI-YFP-Fw	AACTGCAGGTTTCTAAAGGTGAAGAATTATTC
NheI-YFP-Rv	CTAGCTAGCTTATTTATATAATTCATCCATACC
Pmpp1-BamHI-Rv	CGGGATCCTATATGAAATAAATGAAGTAAGTAAGAAAG
EcoRI-Ptdh3-Fw	CCATGATTACGAATTCACACGTAACCGAAT
NotI-Ptdh3-Rv	GTTGAATTAGCGGCCGCTTTGTTTTATTTGAAGAAGTTTTT
NEB-Ptdh3-Mpp1YFP-Fw	CAAATAAAACAAAGCGGCCGCATGACAACAAC
NEB-Ptdh3-Mpp1YFP-Rv	ACTTGTTGAATTAGCGGCCGCTTATTTATATAATTCATCCATACCTAAAG
Inverse-pTDH3eI-Fw	GCTAATTCAACAAGTTGTATCTTTTTTTAC
Inverse-pTDH3eI-Rv	CGCTTTGTTTTATTTGAAGAAGTTTTTG

TABLE S4 Continued

Designation	DNA sequence (5'-3')
NotI-AOD1-Fw	AAGGAAAAAAGCGGCCGCATAATGGCTATCCCAGAAGA
NotI-AOD1-Rv	AAGGAAAAAAGCGGCCGCTTAATAACGAGCAGCACCAG
NotI-DAS1-Fw	AAGGAAAAAAGCGGCCGCAAAATGGCTCTCGCAAAAGC
NotI-DAS1-Rv	AAGGAAAAAAGCGGCCGCATTAAAGTGTCTGCTGTAAATC
ChIP-AOD1-Fw	CCCAGCTTTTCAATTTAATAAAATAGCC
ChIP-AOD1-Rv	GATAGTAAATATAGTAAAATGTGATATGGG
ChIP-DAS1-Fw	ATATTTGGTG GACCTCTCAG TTGCATT
ChIP-DAS1-Rv	AGTCCACTTGACTGAAGACTACGCTAAT
ChIP-FDH1-Fw	TGTTACAATTGTCACAATTCTTGGATATAC
ChIP-FDH1-Rv	AACATCTGACTAGTATTACCATAAATGTAC
ChIP-MPP1-Fw	TGTTTCTGGTTTGTTCAACCCCTCA
ChIP-MPP1-Rv	GCGTATATTCAGATTTAGAGAAAGGTTA
ChIP-ACT1-Fw	CCACTGAGTTCCTTCTTTCTGTTT
ChIP-ACT1-RV	AGAATCTGGGAGGAAAGAATAGAGA
RT-MPP1-Fw	TTCACCACCTTCACTTTCAGG
RT-MPP1-Rv	GCATGCCATATCTGTGATTTTG
RT-AOD1-Fw	TGCTGCTCCAGATTTCGATCC
RT-AOD1-Rv	GGTTCACCAGCAAAACATTCC
RT-DAS1-Fw	TGCAGCCCCAGCATTAAGAA
RT-DAS1-Rv	GCAATAGGTTGATGTGTTGGTCC
RT-FLD1-Fw	GACCGATGGTGGTTGTGATTTC
RT-FLD1-Rv	GCGGCAACACCAATGATAACAG
RT-ACT1-Fw	TTGTCCCAATTTACGCTGG
RT-ACT1-Rv	CAGCAGTGGTGGAGAAAGTG
Southern-URA3-Fw	TGTTGATCTCACCACAACCAA
Southern-URA3-Rv	GCATTCCAACCTGCTTTCAT
Infusion-KpnI-Pmit1-pIB1-Fw	5'-CTATAGGGCGAATTGGGTACCCCTCTTGATGGCTTTGCAGGAAAC-3'
Infusion-BamHI-MIT1-pIB1-Rv	5'-CGAGACTAGTGGATCCTTCTTCAACATTCCAGTAGTC-3'
Inf-Pgap-MIT1-Fw	5'-GGTACCCGGGGATCCACTAG-3'
Inf-Pgap-MIT1-Rv	5'-GGCTGCGGTACTCATGAATTCTTGATAGTTGTTCAATTGATTG-3'
Inf-MIT1-pIB1arg-Fw	5'-ATGAGTACCGCAGCCCCAATCAAGGAAGAAAGCCAATTTGC-3'
Inf-KpnI-pIB1arg-Rv	5'-GGATCCCCGGGTACCCAATTC-3'
Inf-pIB1his-Fw	5'-GACTGCAGGCATGCAAGCTTCTTAGACATGACTGTTCCTC-3'
Inf-pIB1his-Rv	5'-AGTGAGTCGTATTACACTGGCCGTCGTTTTACAAC-3'
Inf-MIT1FLAG-Fw	5'-GTAATACGACTCACTATAGGGCGAA-3'
Inf-MIT1FLAG-Rv	5'-TGCATGCCTGCAGTCATAAATCA-3'
RT-KpMIT1-Fw	5'-GACTAATGACGATGAACTAAG-3'
RT-KpMIT1-Rv	5'-TGCTGTTGTTGGTAGAAT-3'
RT-KpAOX1-Fw	5'-TTTCGAAGGTCCAATCAAGG-3'
RT-KpAOX1-Rv	5'-GTTCAGCACCGTGAGCAGTA-3'
RT-KpDAS-Fw	5'-GGTGACGAGTTAGTAAAGAAC-3'
RT-KpDAS-Rv	5'-CCTCTAACACGAGAAAGGAAC-3'
RT-KpACT1-Fw	5'-TCCGTATGGATCGGTGGTTC-3'
RT-KpACT1-Rv	5'-TTGAGGTGCACAATGGATGG-3'
Kpmit1d-Fw	5'-TTTGATCGAAGCGAGCTACAAGTCC-3'
Kpmit1d-Rv	5'-ACTCCTTCATCCTCCGGTCTTTG-3'

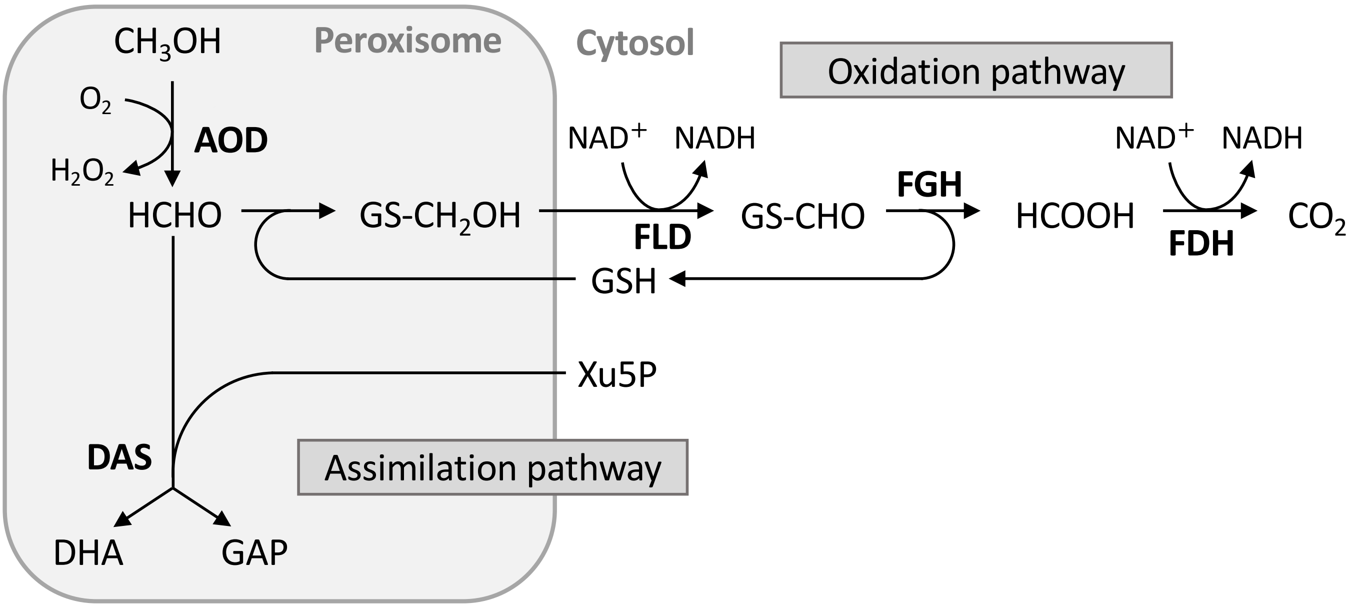

FIG S1 Enzymes associated with methanol metabolism. The pathway of methanol metabolism. Methanol is first oxidized by alcohol oxidase (AOD) to generate formaldehyde and H_2O_2. Formaldehyde, a central intermediate of the methanol metabolism pathway, is positioned at the branch point of the assimilatory pathway and the dissimilatory pathway. In the assimilatory pathway, formaldehyde is fixed by dihydroxyacetone synthase (DAS) to xylulose 5-phosphate (Xu5P) to produce dihydroxyacetone (DHA) and glyceraldehyde 3-phosphate (GAP), which are then consumed to synthesize cell components. AOX and DAS are both localized in peroxisomes. In the dissimilatory pathway, formaldehyde is further oxidized to CO_2 by glutathione (GSH)-dependent formaldehyde oxidation pathway including formaldehyde dehydrogenase (FLD), *S*-formylglutathione hydrolase (FGH) and formate dehydrogenase (FDH). Nucleotide accession numbers of genes encoding AOD, DAS, FLD, FGH, and FDH in *C. boidinii* are M81702, AF086822, AB085186, AB104827, and AF004096, respectively.

```
CbMpp1          MTTTSCDLLVSPIHQSSITSVMSDSKSSPRSKNIDSLKSINTSNNIPSPSTNPIM---KN     57
OpMpp1          -----------------------------------MSISRDDDHPRPK----------     13
KpMit1          MS------TAAPIKEE---------------SQFAHLTLM--NKDIPSNAKQAKSKVSAA     37
                                                       :  ..: *

CbMpp1          RKGRRCSKIKKEKQNLTPPAKRRVRTGCLTCRRKHKKCDENRNPKCDFCTSKGLDCIWPE     117
OpMpp1          ------RARSGKPKKETPPAKRRVRTGCLTCRRKHKKCDENR-PKCDFCTAKGLECVWPE     66
KpMit1          PAKTGSKSAGGSGNNNAAPVKKRVRTGCLTCRKKHKKCDENRNPKCDFCTLKGLECVWPE     97
                           . :: : *.*:**********:********* ******* ***:*:***

CbMpp1          NVKKNVFVNNSLKDFQFDKKSSIHSNINTNHTTTNTTTTTTTASNNNNNNNQLDYNYSD     177
OpMpp1          EVKKNVFVNNSFKDFCFSQSKHRDRFRKP------------------------TDFDLSS     102
KpMit1          NNKKNIFVNNSMKDFLGKKTVDGADSLNLAVNLQQQQSS-NTIANQSLSSIGLESFGYGS     156
                : ***:*****:***  .:.       :                          .:. ..

CbMpp1          PIINHQLRHSIDYTDLIIGGGNNNNSNIL--------DDTSSAFQYKRRKTSFLPESASL     229
OpMpp1          ---------SLSPLDYHLGPDDPR-----------------------TRKKAYSLDVA-D     129
KpMit1          GI-----KNEFNFQDLIGSNSGSSDPTFSVDADEAQKLDISNKNSRKRQKLGLLPVSNAT     211
                         .:.  *   . ..                          :* .

CbMpp1          NLSEGFDGISNKLSRNLSSSNNNSNNNNNDI-STNPSSAPSSTTNTTTSTTSNNNNISSS     288
OpMpp1          HYLSAFDGYSRKNE----------------Y-LDAPLTTAYPL-------------LETS     159
KpMit1          SHLNGFNGMSNGKSHSFSSPSGTNDDELSGLMFNSPSFNPLTVN-----DSTNNSNHNIG     266
                   ..*:* *.  .                     *                     . .

CbMpp1          LLPFNNISTGISASPSSISLSKFVFDQSSNFFGNNNSTSNNRYSSNAILGLSPSNGLLGS     348
OpMpp1          ESPLSGLFNAPQELPSQLTRTPSFGASSLSYFDTLQ-----SYSMNPS------------     202
KpMit1          LSPMSCLFSTVQEASQKK-------------HG--NSSRHFSYPSGP-------------     298
                  *:. : .  .   ..              ..  :      *  .

CbMpp1          LVSSTDYANNSNSNNTNTSANSANTNSLLYNKDEPSASRMTRSSSLNLDLISQSLNNLTS     408
OpMpp1          ------------------------------------------------L--------SS     205
KpMit1          -------------------------EDLWFNEFQKQA------------LTANGENAVQQ     321
                                                 *         .

CbMpp1          NSNKNLLNLNLDYNSNTTSTATTTAPSSVFSSDNINNGNNNNNNNKTSLNDF----EHRN     464
OpMpp1          GSNDKI------------ST-----ASSVS-SD---------MSLAKKDADF----EHRY     234
KpMit1          GDDASK--------NNTAIPKDQSSNSSIFSSRSSAASSNS-GDDIGRMGPFSKGPEIEF     372
                ..: .                     **:  *           .       *    * .

CbMpp1          SFNTFDNTNTKQQTTTSSNFIPQNFEFNDTLNNFITFLISNKSDLLNNLNDNNNNSNDNN     524
OpMpp1          SFAEPQ---------KKSKFGLDYYDLPNTLNDLMCLFIATKDSTSGS------------     273
KpMit1          NYDSFLES------LKAESPSSSKYNLPETLKEYMTLSSSHLNSQHSDTLANGTNGNYSS     426
                .:             .  ..   . ::: :**:: : :  :  ..  ..

CbMpp1          DIDNDNIDNENNFKSKINKFLINNNYSNNFNNIIKPEISDEEMMKLMGFYIDNFSNFLNI     584
OpMpp1          KLP----NNETIFE-----------IFTDKSTMEAPSIDDHEMMLLMETYIDQIAHFLNK     318
KpMit1          -------TVSNNLSLSLNS-----FSFSDKFSLSPPTITDAEKFSLMRNFIDNISPWFDT     474
                         .. :.             .:  .:  * * * * : **  :**::: :::

CbMpp1          LIGEESIKNFSTLLISLSKDFEPLKYCLLALSSRYDQ-----TNNGNTFKYYKYSIDLLI     639
OpMpp1          SD-ETQCNIFMTKIPELAHRFPSLKYAILGLASRHLEKVRSDYSGEKTLQYYTYSLQQLS     377
KpMit1          FD---NTKQFGTKIPVLAKKCSSLYYAILAISSRQRERIKKE-HNEKTLQCYQYSLQQLI     530
                     . : * * :  *::    * *.:*.::**  :       . :*:: * **:: *

CbMpp1          KNSVEILTLNKSIDDEISTILNKILITCIMLTIFEVLSSNQSLWKSKLKICGCLLKNFNF     699
OpMpp1          MC----------LNS--DKNTLEVVATCVLLCYFEILSTNPDSWHSRLENCGSMLKVCHI     425
KpMit1          PTV-------------QSSNNIEYIITCILLSVFHIMSSEPSTQRDIIVSLAKYIQACNI     577
                                 ..   : : **::*  *.::*:: .  :. :   .  ::  ::

CbMpp1          NI-LSVDPIKRGLFLCFARMDFGSVLISEDSTCLNSKDWFPIDISYNHGIKLL-------     751
OpMpp1          NA-RSETELEKALFWSFAALDIGCCAVGDRKTVVRPEDWFGTDAPSS-----P-------     472
KpMit1          NGFTSNDKLEKSIFWNYVNLDLATCAIGEESMVIPFSYWVKETTDYKTIQDVKPFFTKKT     637
                *   *   :::.:*  :. :*:.   :.: .  :  . *.      .

CbMpp1          ------KEQGSSVYYIMIICSKIFNLISSIDKDLDFDNEWVLIWDELKDWEKNKDDCMLQ     805
OpMpp1          ------TETGTALQSMLYLSCQIFQLVSSNDDQSEFDSCWKSLWKQLGDWERARSSN-MR     525
KpMit1          STTTDDDLDDMYAIYMLYISGRIINLLNCRDAKLNFEPKWEFLWNELNEWELNKPLTFQS     697
                         .     :: :. :*::*:.. * . :*:  *  :*.:* :**  :

CbMpp1          CFNYKRN------DSFPEILYPNSSAVISNQLFHMCCILMIQNKPRLVKISNSNNTFKEF     859
OpMpp1          GFCFKRD------NSFPETLFSSSTALCANQLFHTCCILMLQTKPRLVKLHEPEKQER--     577
KpMit1          IVQFKANDESQGGSTFPTVLFSNSRSCYSNQLYHMSYIILVQNKPRLYKIPFTTVSASMS     757
                 . :* :      .:**  *: .* :  :***:* . *:::*.**** *:

CbMpp1          SSTPMQLNSSESSDTSVTSS-TSSTSSSNDSLFQSAVTNNCTSNSPPSLSGSSSPIPNHS     918
OpMpp1          ---------------------------TPDTLFQSAIKTEQG------------------     592
KpMit1          S--PSDNK----AGMSASSTPASDHHASGDHLSP--------RSVEPSLSTTLSPPP---     800
                                           : * *

CbMpp1          TTTKSSNLPITTTISSVDNRKTCQSKSQIWHAKQILGINLCNMKRENCKNNGCFFLSLQC     978
OpMpp1          ---------------ESVSTSSLMSRSQIWHAKQIIGINVCNMKSASSRSLGCQILSLQA     637
KpMit1          ----------------NANGAGNKFRSTLWHAKQICGISINNNHN---SNLAAKVNSLQP     841
                                  .      :* :****** **.: * :     . .. . ***

CbMpp1          IWIAGKLISSSHEHSIVLQILEEMESWYGIDTKWRSQMLVDFWNSET  1025
OpMpp1          IWIAGKLISSAHEHSIILQLLQRLESWSSMSMDWREKQLTEFWKREC  684
KpMit1          LWHAGKLISSKSEHTQLLKLLNNLECATGWPMNWKGKELIDYWNVEE  888
                :* *******  **: :*::*:.:*.  .   .*: : * ::*: *
```

FIG S2 Amino acid sequence alignment of CbMpp1, OpMpp1 and KpMit1. Sequence alignment was generated using CLUSTALW. The conserved DNA binding region in all methylotrophic yeasts is highlighted with grey boxes. Sequence conservation is represented using different symbols as follows: “*”: identical or conserved in all sequences in the alignment; “:”: conserved substitutions; “.”: semi-conserved substitutions. The identity and similarity values between CbMpp1 and OpMpp1 are 24% and 34%, respectively, and those between CbMpp1 and KpMit1 are 22% and 35%, respectively. The conserved DNA binding region (76-132 a.a.) shows 89% identity and 94% similarity to the zinc finger domain of OpMpp1 (26-81 a.a.) and KpMit1 (56-112 a.a.), respectively.

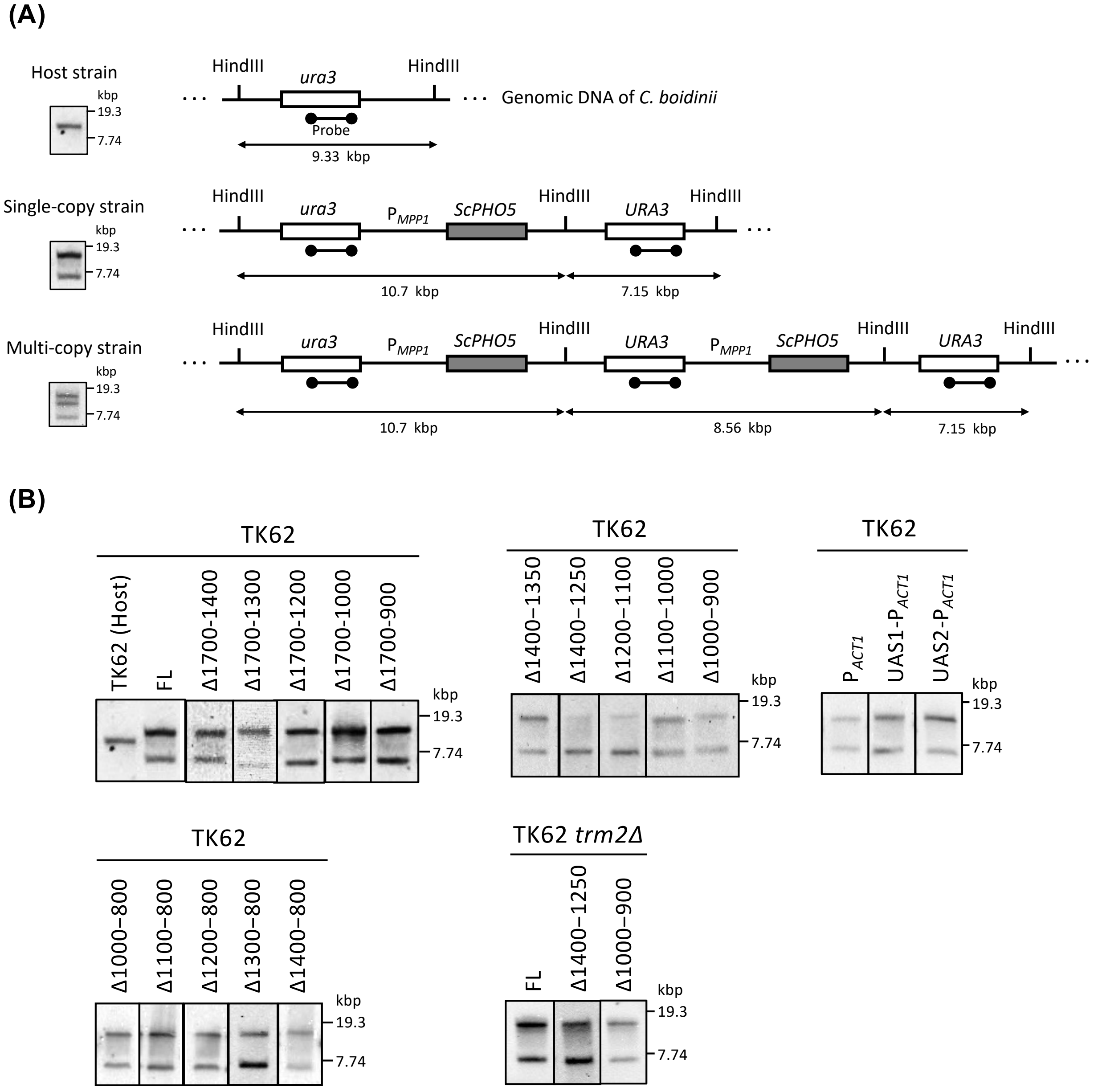

FIG S3 Scheme and preparation of acid phosphatase assay for truncated *CbMPP1* promoter activity measurement. (A) The structure of *URA3* locus in genomic DNA of *C. boidinii* host strain (TK62), single-copy inserted strain and multi-copy inserted strains for Southern blot analysis. The theoretical fragment size from genomic DNA by HindIII digestion has been indicated. The labelled DNA probe was designed against the *URA3* region and prepared for Southern blot analysis. The plasmid pMPU1 was introduced in TK62 or TK62 *Cbtrm2Δ* strain, resulting in a single-copy-inserted or muti-copy-inserted strain. A single band signal was detected from the host strain in Southern blot analysis. Two band signals were detected from the single-copy strain, and three band signals were detected from the multi-copy strain. (B) Southern blot analysis of the strains used in the acid phosphatase assay for confirming single-copy insertion of the plasmid. Cells were cultured in YPD media for 24 h and genomic DNA was extracted.

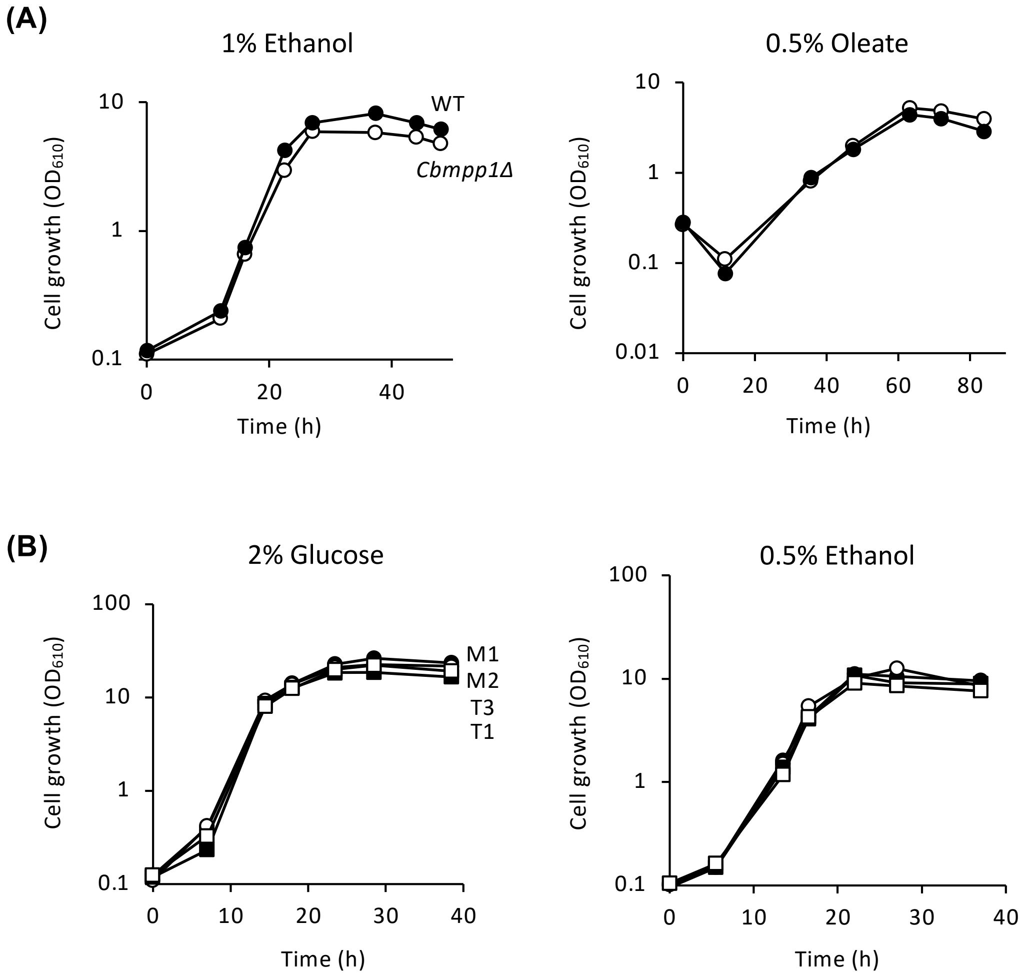

FIG S4 Growth of the *C. boidinii* strains on various carbon sources. (A) Cell growth of wild type (filled circles) and *Cbmpp1Δ* (open circles) strains on various carbon sources. Cells were pre-cultured in SD medium and then grown in YNB medium containing 1% ethanol or 0.5% oleate. (B) Cell growth of strains overexpressing or constitutively expressing *CbMPP*1 was measured on YNB media containing 2% glucose or 0.5% ethanol. Symbols; strains M1 (filled circles), M2 (open circles), T1 (filled squares) and T3 (open squares).

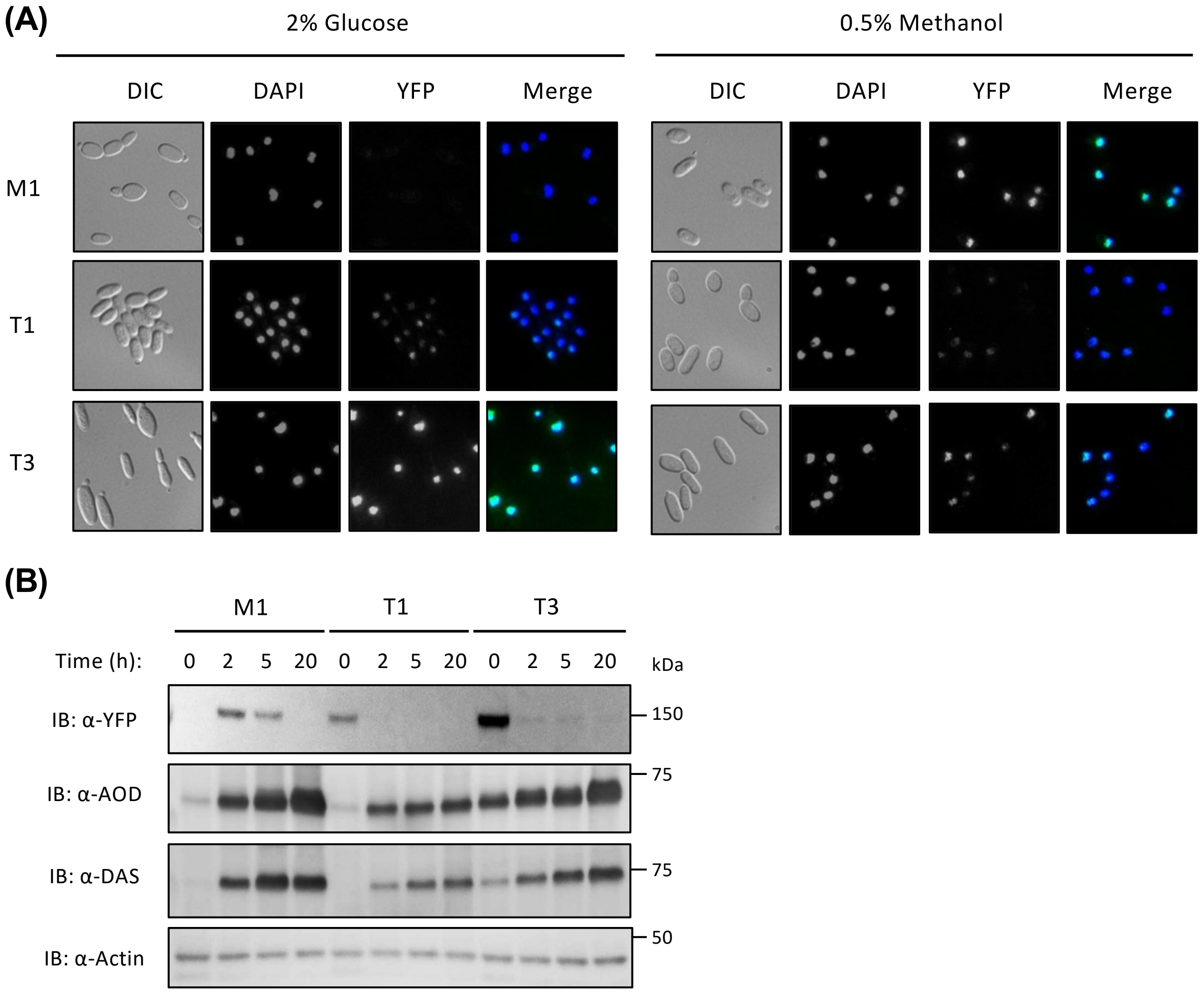

FIG S5 Subcellular localization of CbMpp1-YFP and protein levels of CbMpp1-YFP, AOD and DAS in strains M1, T1 and T3. (A) Fluorescence microscopy of CbMpp1-YFP in strains M1 (control), T1 and T3. Cells were shifted from SD to SM medium containing 0.5% methanol for 5 h. Subsequently, they were treated with 70% ethanol for 30 min and stained with 50 μg/L DAPI for 20 min. DAPI was used to stain the cell nucleus. DIC, differential interference contrast. The scale bars correspond to 5.0 μm. (B) Immunoblot analysis of CbMpp1-YFP (α-YFP), AOD (α-AOD) and DAS (α-DAS) protein levels in strains M1 (control), T1 and T3. Actin was blotted as a loading control (α-Actin). Cells were shifted from glucose medium (SD) to 0.5% methanol medium (SM) for 20 h and collected at the indicated time points. The samples were loaded on 7.5% acrylamide SDS-PAGE gel (SuperSep Ace). Molecular weights of the protein size marker are indicated.

References

1. Sakai Y, Tani Y. 1992. Directed mutagenesis in an asporogenous methylotrophic yeast: cloning, sequencing, and one-step gene disruption of the 3-isopropylmalate dehydrogenase gene (*LEU2*) of *Candida boidinii* to derive doubly auxotrophic marker strains. *J Bacteriol* 174:5988-5993.
2. Yurimoto H, Komeda T, Lim CR, Nakagawa T, Kondo K, Kato N, Sakai Y. 2000. Regulation and evaluation of five methanol-inducible promoters in the methylotrophic yeast *Candida boidinii*. *Biochim Biophys Acta* 1493:56-63.
3. Sasano Y, Yurimoto H, Yanaka M, Sakai Y. 2008. Trm1p, a $Zn(II)_2Cys_6$-type transcription factor, is a master regulator of methanol-specific gene activation in the methylotrophic yeast *Candida boidinii*. *Eukaryot Cell* 7:527-536.
4. Nakagawa T, Ito T, Fujimura S, Chikui M, Mizumura T, Miyaji T, Yurimoto H, Kato N, Sakai Y, Tomizuka N. 2004. Molecular characterization of the glutathione-dependent formaldehyde dehydrogenase gene *FLD1* from the methylotrophic yeast *Pichia methanolica*. *Yeast* 21:445-453.
5. Sakai Y, Akiyama M, Kondoh H, Shibano Y, Kato N. 1996. High-level secretion of fungal glucoamylase using the *Candida boidinii* gene expression system. *Biochim Biophys Acta* 1308:81-87.
6. Yurimoto H, Yamane M, Kikuchi Y, Matsui H, Kato N, Sakai Y. 2004. The pro-peptide of *Streptomyces mobaraensis* transglutaminase functions in *cis* and in *trans* to mediate efficient secretion of active enzyme from methylotrophic yeasts. *Biosci Biotechnol Biochem* 68:2058-2069.
7. Inoue K, Ohsawa S, Ito S, Yurimoto H, Sakai Y. 2022. Phosphoregulation of the transcription factor Mxr1 plays a crucial role in the concentration-regulated methanol induction in *Komagataella phaffii*. *Mol Microbiol* 118:683-697.
8. Ohsawa S, Nishida S, Oku M, Sakai Y, Yurimoto H. 2018. Ethanol represses the expression of methanol-inducible genes via acetyl-CoA synthesis in the yeast *Komagataella phaffii*. *Sci Rep* 8:18051.
9. Takeya T, Yurimoto H, Sakai Y. 2018. A *Pichia pastoris* single-cell biosensor for detection of enzymatically produced methanol. *Appl Microbiol Biotechnol* 102:7017-7027.
10. Sakai Y, Saiganji A, Yurimoto H, Takabe K, Saiki H, Kato N. 1996. The absence of Pmp47, a putative yeast peroxisomal transporter, causes a defect in transport and folding of a specific matrix enzyme. *J Cell Biol* 134:37-51.
11. Tani Y, Sakai Y, Yamada H. 1985. Production of formaldehyde by a mutant of methanol yeast, *Candida boidinii* S2. *J Ferment Technol* 63:443-449.

12. Sakai Y, Kazarimoto T, Tani Y. 1991. Transformation system for an asporogenous methylotrophic yeast, *Candida boidinii*: cloning of the orotidine-5'-phosphate decarboxylase gene (*URA3*), isolation of uracil auxotrophic mutants, and use of the mutants for integrative transformation. *J Bacteriol* 173:7458-7463.
13. Sasano Y, Yurimoto H, Kuriyama M, Sakai Y. 2010. Trm2p-dependent derepression is essential for methanol-specific gene activation in the methylotrophic yeast *Candida boidinii*. *FEMS Yeast Res* 10:535-544.
14. Oda S, Yurimoto H, Nitta N, Sakai Y. 2016. Unique C-terminal region of Hap3 is required for methanol-regulated gene expression in the methylotrophic yeast *Candida boidinii*. *Microbiology* 162:898-907.
15. Nakagawa T, Mukaiyama H, Yurimoto H, Sakai Y, Kato N. 1999. Alcohol oxidase hybrid oligomers formed *in vivo* and *in vitro*. *Yeast* 15:1223-1230.
16. Sakai Y, Nakagawa T, Shimase M, Kato N. 1998. Regulation and physiological role of the *DAS1* gene, encoding dihydroxyacetone synthase, in the methylotrophic yeast *Candida boidinii*. *J Bacteriol* 180:5885-5890.
17. Sakai Y, Koller A, Rangell LK, Keller GA, Subramani S. 1998. Peroxisome degradation by microautophagy in *Pichia pastoris*: identification of specific steps and morphological intermediates. *J Cell Biol* 141:625-636.
18. Sakai Y, Yurimoto H, Matsuo H, Kato N. 1998. Regulation of peroxisomal proteins and organelle proliferation by multiple carbon sources in the methylotrophic yeast, *Candida boidinii*. *Yeast* 14:1175-1187.
19. Sears IB, O'Connor J, Rossanese OW, Glick BS. 1998. A versatile set of vectors for constitutive and regulated gene expression in *Pichia pastoris*. *Yeast* 14:783-790.
20. Tamura N, Oku M, Sakai Y. 2010. Atg8 regulates vacuolar membrane dynamics in a lipidation-independent manner in *Pichia pastoris*. *J Cell Sci* 123:4107-4116.
